# Home gardens of Central Asia: Reservoirs of diversity of fruit and nut tree species

**DOI:** 10.1371/journal.pone.0271398

**Published:** 2022-07-28

**Authors:** Barbara Vinceti, Marlène Elias, Rashid Azimov, Muhabbat Turdieva, Sagynbek Aaliev, Farhod Bobokalonov, Evgeniy Butkov, Elmira Kaparova, Nurullo Mukhsimov, Svetlana Shamuradova, Kubanichbek Turgunbaev, Nodira Azizova, Judy Loo

**Affiliations:** 1 Bioversity International, Rome, Italy; 2 Bioversity International, Tashkent, Uzbekistan; 3 Kyrgyz National Agrarian University named after K. I. Skryabin, Bishkek, Kyrgyzstan; 4 Institute of Horticulture and Vegetable Growing of Tajik Academy of Agricultural Sciences, Dushanbe, Tajikistan; 5 Republican Scientific and Production Center of Ornamental Gardening and Forestry, Tashkent, Uzbekistan; 6 National University of Uzbekistan named after Mirzo Ulugbek, Tashkent, Uzbekistan; Institute for Horticultural Plants, China Agricultural University, CHINA

## Abstract

Central Asia is an important center of origin for many globally valued fruit and nut tree species. Forest degradation and deforestation are cause for concern for the conservation of these valuable species, now confined to small remnant populations. Home gardens have the important function of sustaining household food consumption and income generation, and can potentially play a critical role in conserving diversity of fruit and nut trees. These systems have been very poorly documented in the scientific literature. This study contributes to filling this gap by describing the diversity of fruit and nut trees in home gardens of Kyrgyzstan, Uzbekistan, and Tajikistan, examining their dynamic flow of planting material and its sources, understanding their future prospects, and looking at significant differences between the three countries. Home gardens show a similar portfolio of the most abundant tree species (apple, apricot, walnut, pear, and plum). Although the diversity of tree species and varieties recorded is significant, small population sizes can limit future possibilities for this diversity to thrive, given the pressure on natural stands and on habitats where the preferred species are found. Furthermore, the selection of species and varieties to be planted in home gardens is increasingly influenced by market opportunities and availability of exotic material. Some of the most abundant tree species recorded are represented largely by exotic varieties (apple, pear), while others (e.g., apricot, walnut, plum) are still mainly characterized by traditional local varieties that are not formally registered. Home gardens continue to play a critical role in rural livelihoods and in national economies, and many rural inhabitants still aspire to maintain them. Thus, home gardens should be integrated in national research and extension systems and closely linked to national conservation efforts. Changes and possible declines in the diversity they host, their health status, and resilience should be carefully monitored.

## Introduction

Central Asia is a center of diversity for many globally valued fruit and nut tree species, including apple (*Malus* spp.) [1], apricot (*Prunus armeniaca*) [2], walnut (*Juglans regia*) [3, 4], pear (*Pyrus* spp.), plum (*Prunus* spp.), almond (*Prunus dulcis*), pomegranate (*Punica granatum*), and grape (*Vitis* spp.). Wild relatives of these and other valued species still grow spontaneously in forests throughout the region, often used as rootstock for trees planted in home gardens and orchards or in restoration projects, as they are better adapted to local condition and more resistant to biotic stresses, such as pests and diseases, than some improved, “modern” varieties. Many of these species are harvested by local people in the high-altitude forests of Kyrgyzstan, Uzbekistan, and Tajikistan, where they grow. For most forest dwellers, fruit and nut species harvested in the wild are the main source of income [5] and important nutritional resources.

Yet, forest degradation and deforestation due to grazing and tree logging are causing concerns for the conservation of these valuable species, which are now confined to a limited number of small remnant populations. The main causes of deforestation, particularly pronounced in Kyrgyzstan and Tajikistan, include illegal cutting, conversion of forest land to agriculture, and harvesting of fuelwood [6]. Moreover, overgrazing and overharvesting in fruit and nut forests—linked to the difficult socio-economic conditions of local populations—is compromising the long-term resilience of these systems [7]. Ecological studies focusing on fuelwood collection and restoration in the Pamirs (Tajikistan) [8, 9], and assessing changes in composition, structure, and soil conditions in the walnut-fruit forests of Kyrgyzstan [10–14], show growing erosion of globally significant plant genetic diversity.

In addition to trees in forests, tree species cultivated in home gardens play an important role in sustaining genetic diversity, household consumption, and income generation. Home gardens, also referred to as forest gardens, are small land areas located near the homestead where multiple crops are grown, usually in multiple layers. These systems are critical for producing food and other essential livelihood products within larger production systems [15, 16]. A large share of food products is provided by home gardens in Uzbekistan (78.5%) based on official statistics on the agricultural sector [17] and in Tajikistan (93%) based on information from the Central Asian Bureau for Analytical Reporting [18].

The role of home gardens in food and income security has increased during the COVID-19 pandemic, which has restricted international and local travel and seasonal labor migration. Household economies in Kyrgyzstan, Tajikistan and Uzbekistan were significantly affected, as their main source of income had been seasonal labor migration to neighboring countries, such as Russia and Kazakhstan. As a consequence, in April 2020, the governments of Uzbekistan and Tajikistan declared support to cultivate food crops in home gardens, and the will to better connect products grown in household plots with domestic and foreign markets [19].

Yet, despite their central and continued importance to feed families and contribute to their livelihoods, and their critical role in conserving important tree genetic resources in the region, home gardens in Central Asia have been thinly documented in the English-language scientific literature. The main scientific reviews of these management systems (e.g., [20, 21]) are dated, and do not incorporate studies from this region. Moreover, the literature on home gardens has a privileged focus on cultivated species other than trees (e.g. vegetables, herbs), and scant attention has focused on the interconnections between home gardens and forests, on the impact of local management practices on home garden diversity, and on the role these practices may play in maintaining the dynamic evolution of plant genetic diversity.

To contribute to filling this gap, and to assess the potential role of these systems in conserving key fruit and nut tree resources, this study documents plant diversity hosted in home gardens in Kyrgyzstan, Uzbekistan, and Tajikistan. More specifically, the study addresses the following questions:

1. What level of plant interspecific and intraspecific diversity, with emphasis on trees, is hosted in the home gardens of the three study countries?
2. What are the sources of the material planted in home gardens?
3. What factors guide the sourcing of planting material used in home gardens, particularly from the forest?
4. Is planting home garden material in the forest a common practice, and, if so, what motivates this practice?
5. Are there significant differences among the three countries with respect to the above aspects?

We first turn to a brief review of the English-language literature on the significance of home gardens for conserving plant genetic resources, with a focus on the Central Asian region, to contextualize the importance of the study.

### Home gardens and plant genetic resource conservation

Many home gardens harbor unique and rare species and a high genetic diversity of crop plants and their wild relatives. They are centers of experimentation on species domestication and crop improvement, sources of exchanged genetic material, and home to newly acquired germplasm [20, 22]. As such, home gardens are refuges for wild species threatened by deforestation and urbanization.

Home gardens reflect a close interaction between human cultures and nature. Within these systems, humans carve diversified niches for different plants to grow in small multi-functional and complex structures [21]. The composition and structure of home gardens are dynamic and influenced by the socioeconomic circumstances and cultural background of the households that manage them [23]. In Kyrgyzstan, Tajikistan, and Uzbekistan, rural inhabitants have conserved a large and diverse range of domesticated plants and several landraces of useful species, such as apple, apricot, grapevine, pomegranate, fig, and pear among others, with unique characteristics in their home gardens. These are generally of a smaller size and host a lower number of tree individuals than orchards, where trees are counted in the order of hundreds.

Rich local ecological knowledge underpins farmers´ skills and practices related to fruit tree management in home gardens and orchards in Central Asia in relation to the choice of species, varieties and rootstocks, and the layout and row-spacing of trees and shrubs (bushes). This knowledge guides practices such as inter-row tillage, soil health management, watering and fertilizing, pruning, pest and disease management, spring frost and chilling protection, fruit storage and processing, and other biodiversity management practices. Moreover, specialized knowledge allows home gardeners to grow species that are not true to seed and are difficult to root, such as apple, pear, plum, and cherry, for which grafting techniques have become essential for propagation [24–26].

Wiersum [27, 28] suggests that home gardens/forest gardens play a role as a transition from wild to domesticated fruit trees. Likewise, Hughes et al. [29] observe that home gardens favor unplanned hybridization among related fruit tree species, influencing their evolution. Germplasm from the wild is often brought under cultivation in home gardens. For example, some root crop species, such as taro (C*olocasia esculenta*) and yams (*Dioscorea* spp.), have been continually grown in home gardens to renew the vigor of their germplasm for planting in larger fields [30]. Populations of wild or semi–wild fruit trees are frequently maintained near cultivated areas or in forests [31], and hybridized with cultivars grown for fruit production (e.g. wild apple and pear) [32], such that it can be difficult to separate true ‘wild’ forms from cultivated ones [32, 33]. The genetic diversity of wild fruit tree species (e.g., pistachio, walnut, apple, pear, almond) that grow in Central Asian forests helps build resistance to pests in cultivated species and varieties, strengthens their adaptive capacity [34–36], and is the basis for harnessing, through domestication, critical qualitative fruit traits with economic significance, such as storability [37]. Local varieties are genotypes with economic, historical, cultural, and heritage value in particular geographic contexts that exhibit interesting or unique traits and special food uses, but have been used mainly for local consumption as their attributes may limit large-scale commercialization [38].

Conservation of wild fruit and nut tree species is critical to sustain resilience in home gardens and orchards. During Soviet times, very old orchards of different fruit trees and grape vines, bred over many centuries, were largely abandoned and in some cases uprooted to make way for cotton production [5]. More recently, major threats to wild fruit and nut tree resources were reported, especially as a result of the changes in rural economies caused by the break-up of the former Soviet Union in 1991. Pre-existing problems have intensified since independence, particularly the pressure on forest resources due to increased firewood demand (due to a sudden lack of fossil fuels) and construction material, fires, expansion of settlements and agriculture, uncontrolled grazing and collection of non-timber forest products. Potential development of a robust horticultural sector has been constrained, among other factors, by a lack of understanding on the part of decision-makers about the role of wild relatives of valuable plant species in agricultural development, and a lack of consequent measures to document and protect these resources. In addition, the penetration of a market economy has triggered a process of progressive replacement of traditional diversity by modern cultivars and hybrid varieties, partly with support of state policies that promote cultivation of a few market-oriented crops that respond to international demand. As a consequence, fewer farmers are interested in cultivating local landraces [5].

Conservation and sustainable use of wild relatives and landraces in Central Asia must rely on different and complementary approaches *in situ* (in forests), on farm (e.g., in home gardens) and *ex situ* (e.g., in field collections, genebanks) to maintain fruit and nut tree genetic resources in the broader landscape, and to support agricultural development [5, 38]. Understanding the patterns of plant diversity in home gardens, the factors that shape this diversity, and its distribution across species and varieties is critical from a long-term conservation perspective. In addition, the distribution and connectivity of diversity within and across home gardens in the region are crucial for the maintenance of valuable agricultural biodiversity. From a conservation perspective, key concerns for maintaining genetic diversity in home gardens relate to the population sizes of the species grown, selection intensity (i.e. how selective breeders are in deciding how many individuals from the current generation will make offspring for the next generation), and the vulnerability of individual populations to unpredictable events that may destroy unique material.

### Setting and context

Central Asia has a continental climate characterized by high levels of variability, with temperature extremes in the mountains of -45°C and maximum temperatures in desert areas reaching 50°C [39]. Mean annual precipitation has ranged from 60 mm to 1,180 mm across different localities in the region over the last century. Projections suggest that global warming is going to provoke an increased frequency of extreme weather conditions at the regional scale, including an increased drought risk, with associated risks for agricultural production [39, 40].

**Fig 1:**
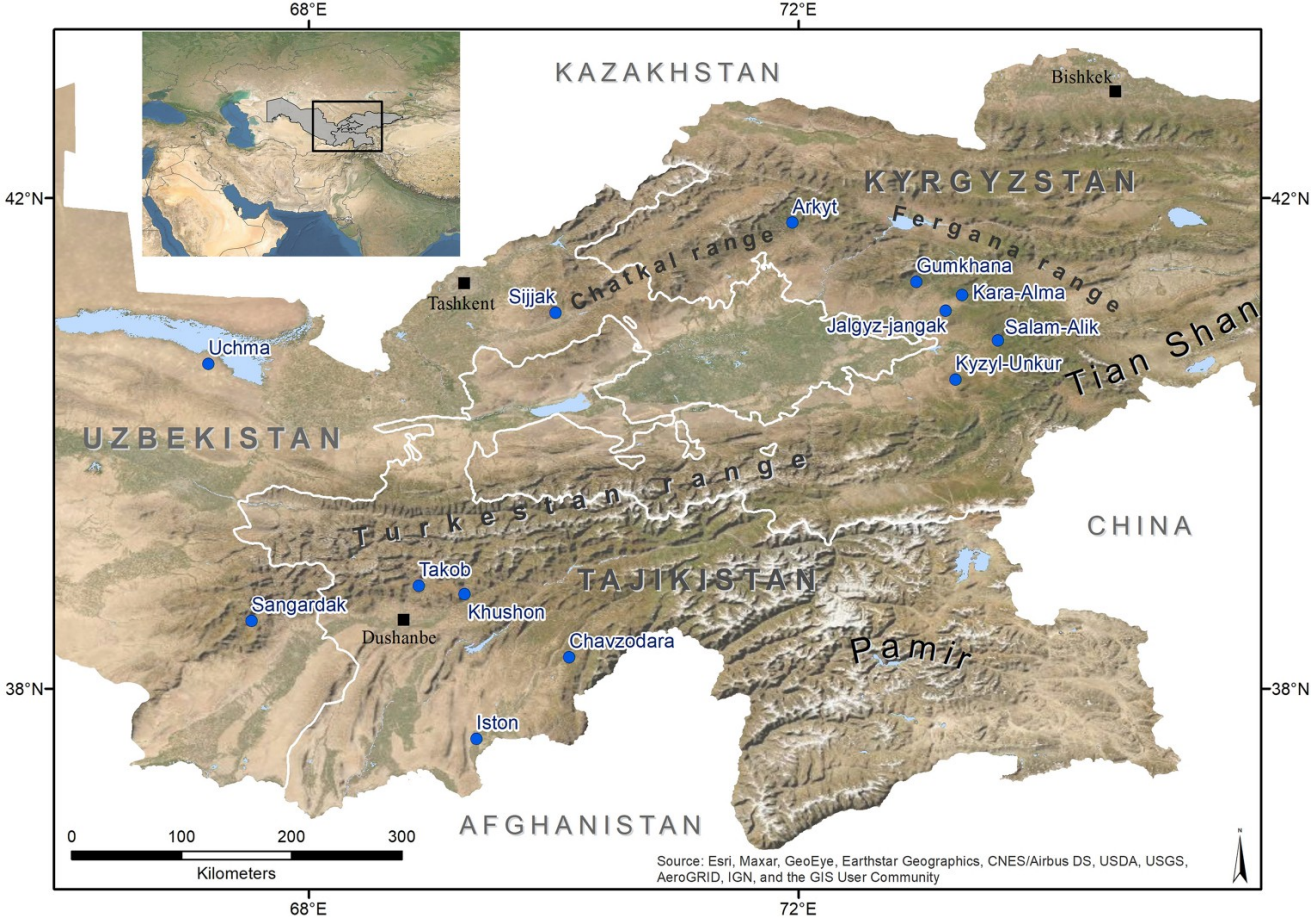
Map of the study sites in the three countries. The blue dots indicate the sites where field work was conducted.

Uzbekistan is largely a flat desert territory (ca. 80%), with mountain peaks reaching > 4,500 meters above sea level (m.a.s.l.) in the eastern part of the country (Fig 1). Its mostly arid continental climate shows an average annual precipitation of 100–200 mm, and water resources are in short supply in most of the country. Kyrgyzstan, home to part of the Pamir and Tien Shan mountains, is an arid and mountainous country. The large majority (90%) of its land sits at more than 1,500 m.a.s.l. Tajikistan is a landlocked country with 93% of the territory represented by mountains and a dense network of rivers.

The tradition of individuals growing their own food and earning income from home gardens has a long history in Russia [41, 42], despite Soviet policies that partly constrained this practice [43]. The establishment of home gardens was encouraged across the Soviet Union to ensure that rural households could produce their own food, to overcome food shortages without having to develop food distribution networks, which were needed to supply food to urban centers [41, 42].

In Kyrgyzstan, traditionally a land of nomadic pastoralists culturally distant from the forest [43], a shift from pastoralism as a main livelihood strategy to sedentary agriculture took place during Soviet times. Home gardens were established at that time, despite the lack of experience of local populations in planting crops, managing a farm, and marketing and selling surplus production [44]. After the fall of the Soviet Union, rural households maintained their small home gardens (called ‘peasant plots’), which continued to play a crucial function for the survival of the rural population [45] and for food supply to urban areas. Today, access to forest products (e.g., nuts, fruits, wood, hay) is regulated by a Participatory Forest Management model, promoted through medium-term leases of forest land (*arendas*) to local households [46].

In Uzbekistan, the Soviet modernization of agriculture consisted of centralized planting of monocultures (mainly cotton) on large, irrigated surfaces. Yet, a high plant diversity was maintained in home gardens, where traditional agriculture was practiced. Home gardens combined fruit and nut trees, grapevines, staple crops, and vegetables for household consumption or market sale. Varieties from these cultivated species were grown, inherited, and exchanged among home gardeners without the involvement of state planning.

In Tajikistan, home gardens gained importance under Soviet rule and in its aftermath. Small-scale farmers have relied on home gardens for household subsistence during and following the civil war (1992–1997), amid land tenure insecurity associated with the slow advance of land privatization following the fall of the Soviet Union [47]. The collectivization of agricultural land for cotton production displaced traditional land uses, including historically widespread orchards and cultivation of mulberry trees [48]. Only small plots of land immediately adjacent to the homes of valley residents were spared from collectivization and left under household management. The cultivation of fruit and nut tree landraces was moved to these small plots, originally reserved for home production but progressively open to market opportunities. More significant income could be generated through selling home garden products at the market than through collective or state farms [49, 50]. Since the breakup of the Soviet Union, all collective and state farms have transitioned to farm laborers’ cooperative enterprises run by citizens with governmental oversight. However, privatization has remained limited to homes and immediately adjacent lands, which Tajiks can manage as they want [47].

Official statistics reported that from 1997–2007, about 60% of Uzbekistan’s agricultural output came from home gardens [51]. In Kyrgyzstan, home gardens represented 22% of total agricultural production in 2007 [52], and over 90% of rural households had home gardens [53]. In Tajikistan, home gardens have been the main source of livelihood for many rural families since the fall of the Soviet Union [47].

## Methods

The study is based on a survey carried out in targeted locations. It involved national research partners in each country and followed their standards. Oral informed consent was obtained at the beginning of each interview by reading a standard introduction to each interviewed person to explain the purpose of the survey and how the data were going to be used. Written informed consent for participation was not required for this study in accordance with the national legislation and institutional requirements in all countries. An Institutional Review Board (IRB) has now been established at Bioversity International but, at the time this research was conducted, such a committee was not in place, therefore we followed the best existing guidelines available at the time.

Field work was carried out between March and September 2016 in six Kyrgyz villages (Salam-Alik, Jalgyz-jangak, Kara-Alma, Gumkhana, Kyzyl-Unkur and Arkyt), four Tajik villages (Takob, Khushon, Chavzodara and Iston), and three Uzbek villages (Sijjak, Uchma and Sangardak) (Fig 1). Village selection was guided by the occurrence of the main tree species of interest (walnut, apple, apricot) and the presence of walnut forests near the villages, to examine the movement of planting material between wild tree populations and home gardens. The number of villages and Forestry Enterprises (state-run forest management units) from each country varied due to differences in the distribution of walnut-fruit tree forests.

A total of 30 respondents—approximately as many men as women–from different households were randomly selected in each village. Local men (n = 206) and women (n = 184) participated individually in semi-structured interviews on the management of fruit and nut trees in their home gardens and orchards (Table 1). Interviews were held in Russian and data were translated into English during data entry. As noted above, Tajikistan and Kyrgyzstan are predominantly mountainous countries with a large share of agropastoralists. Although we refer to ‘farmers’ in this paper, participant livelihoods are also largely based also on pastoral and semi-nomadic activities, particularly in Kyrgyzstan.

**Table 1:** Characteristics of study participants.

		No.	Average age (years)	Frequency (%) of participants in age range (years)		
				18–30	31–55	>55
Kyrgyzstan	Men	89	48	19	46	35
	Women	91	47	15	57	27
Tajikistan	Men	73	44	27	51	22
	Women	47	43	11	70	19
Uzbekistan	Men	44	54	5	48	47
	Women	46	40	17	76	7

The average age of respondents was 46, with most interviewees in the 31–55 age group (Table 1). In each country, respondents were ethnically homogenous: uniformly Uzbek and Tajik in Uzbekistan and Tajikistan, respectively, and almost uniformly Kyrgyz in Kyrgyzstan (fewer than 2% were not ethnically Kyrgyz). Respondents referred to species in their home gardens and orchards based on their local taxonomy. Specialists from national research centers assisted in reviewing the names of varieties to ensure they were correctly spelled. In our inventory of varieties we distinguished between the following categories: forest (material derived from seeds/root suckers harvested in the wild), local (varieties available locally, not widely commercialized across borders, and to which the farmers interviewed could not attribute a name), traditional (varieties available locally and identified through a specific name), exotic (widely commercialized varieties coming from outside the country), and improved local (varieties that originated within the country and have undergone formal breeding). Home gardens with over 300 tree individuals were excluded from our analyses (8 households in total) as not representative and possibly falling in the category of small orchards.

Descriptive statistics were used to analyze the data. A Generalized Linear Model (GLM) with Poisson distribution (Poisson GLM) was used to test whether the number of species was different among countries. A GLM with binomial distribution was used for each species in turn to assess whether the presence of a species (response variable) was correlated with differences in responses by country, gender, and age group of respondents (included in the model, considering age group, country, and gender as covariates). The most predictive model was selected using the Akaike Information Criterion (AIC); the best model would show the lowest value for this indicator. Outliers in terms of the number of trees reported in home gardens were excluded before carrying out statistical analyses. Quality of fit and violation of model statistical assumptions were carried out by graphical inspection of Pearson residuals and computation of Pearson statistics. Analyses were carried out using the statistical software ‘R’ version 4.1.2. All data analyzed and reported are based on participant responses rather than ground-truthing.

## Results

### Characteristics of home gardens

Home gardens in the study sites hosted a diversity of plant species, including trees and shrubs (Table 2). Overall, 18 plant species were recorded across the three countries (Table 2). Of these, 13 were trees and the others were shrub species (*Fragaria* spp., *Rubus* spp., *Rubus idaeus*, *Ribes nigrum*) and a vine (*Vitis* spp.). Improved varieties of apple (§) belonged to *Malus domestica*, while local genotypes/varieties belonged to *Malus sieversii*. The most commonly cultivated species of pears (‡) in the study area were: *Pyrus asiae-mediae* (Popov) Maleev*; Pyrus communis* L.*; Pyrus korshinskyi* Litv.*; Pyrus regelii* Rehder*; and Pyrus bucharica* Litv. Shrubs and vine species were present in a very small fraction of home gardens (ca. 3%), so they are not considered further in our analysis.

**Table 2:** List of species cultivated in home gardens across the study sites in all countries.

Scientific name	Family	Common name
Trees		
*Cydonia oblonga* Mill.	Rosaceae	quince
*Juglans regia* L.	Juglandaceae	walnut
*Malus* spp. (§)	Rosaceae	apple
*Morus alba* L.	Moraceae	mulberry
*Morus rubra* L.	Moraceae	red mulberry
*Prunus armeniaca* L.	Rosaceae	apricot
*Prunus avium* L.	Rosaceae	sweet cherry
*Prunus cerasifera* Ehrh.	Rosaceae	alycha/cherry plum
*Prunus cerasus* L.	Rosaceae	cherry
*Prunus domestica* L.	Rosaceae	plum
*Prunus dulcis* Mill.	Rosaceae	almond
*Prunus persica* L.	Rosaceae	peach
*Punica granatum* L.	Lythraceae	pomegranate
*Pyrus* spp. (‡)	Rosaceae	pear
Other plants (shrubs, vines, small fruit species)		
*Fragaria* spp.	Rosaceae	strawberry
*Ribes nigrum* L.	Grossulariaceae	currant
*Rubus idaeus* L.	Rosaceae	raspberry
*Rubus* spp.	Rosaceae	blackberry
*Vitis* spp.	Vitaceae	grapevine

Across the three countries, the overall mean number of tree and shrub species in home gardens was 4.7, and varied between 1 and 11 species/home garden (Table 3).

**Table 3:** Characteristics of home gardens.

		Kyrgyzstan	Tajikistan	Uzbekistan	Region
Number of species	Mean	4.0	5.6	4.8	4.7
	Standard deviation (SD)	1.7	1.4	1.7	1.8
	Interquartile range	3–5	5–6.75	4–5	3–6
	Max	9	10	11	11
	Min	1	3	2	1
Number of trees	Mean	46.7	56	54.6	51.4
	SD	42.5	43.2	57.4	46.7
	Interquartile range	20–55	27–70.75	22–59	23–61
	Median	33	44	35	36
	Max	245	307	300	307
	Min	5	10	7	5
	No. respondents	178	118	89	385
Surface area (square meters)	Mean	1755.3	1475.5	1368.7	1490.6
	SD	1476.9	1284.3	1008.3	1245
	Interquartile range	1175–1650	700–1600	800–1850	800–1800
	No. respondents	32	109	56	197
Tree density (no. trees/ha)	Mean	266	379	399	345
Years since establishment	Mean	47.3	43.1	46.6	45.9
	SD	14.4	12.6	13.7	13.8
	Max	87	73	81	87
	Min	1	19	19	1

**Fig 2:**
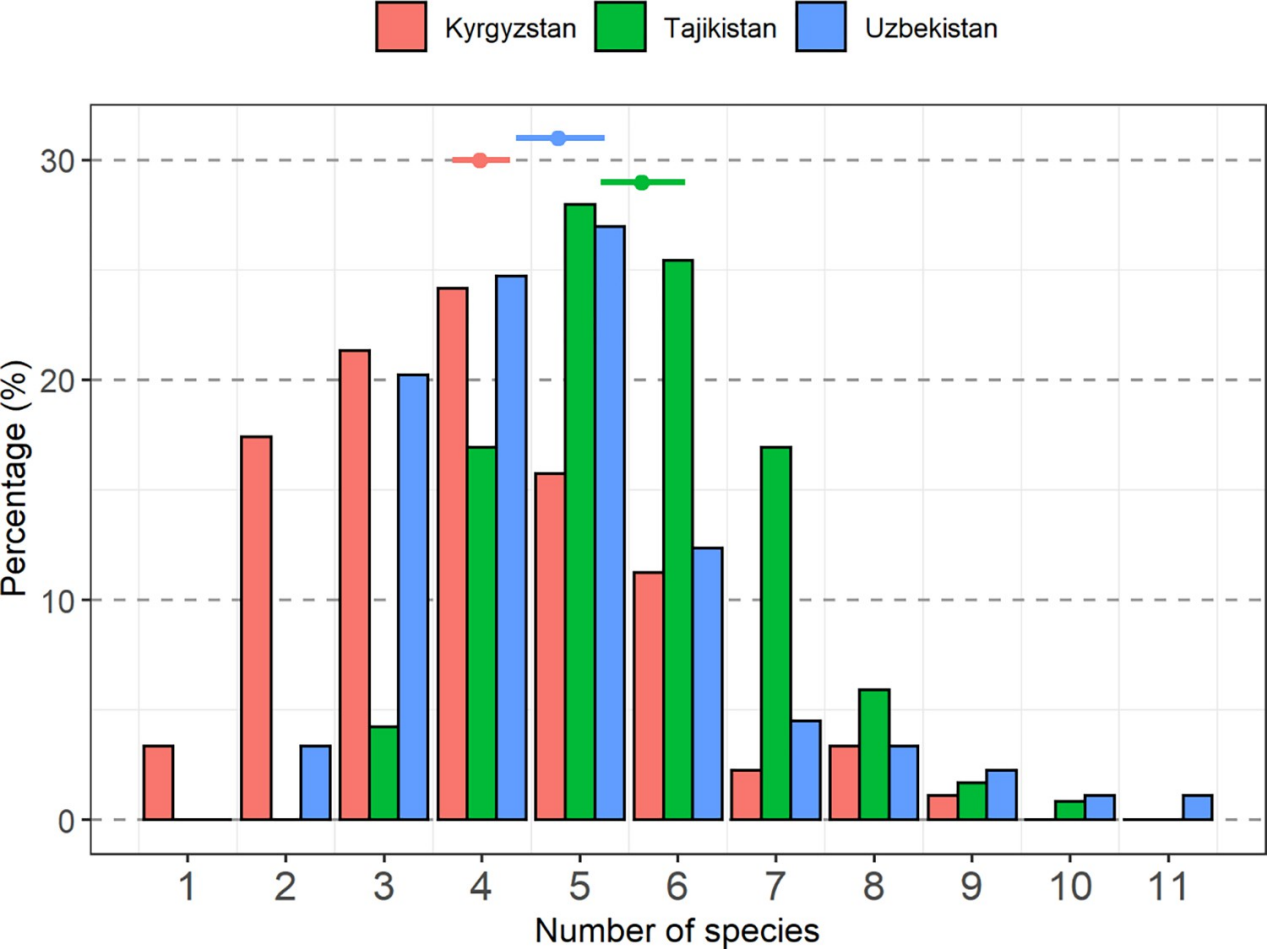
Proportion of participants (% per country) with a given number of plant species in their home garden.

The average number of species was significantly higher in Uzbekistan than in Kyrgyzstan, and significantly higher still in Tajikistan than in Uzbekistan (p<0.0001, based on GLM Poisson) (Fig 2).

The average number of tree individuals in a home garden was 51.4, with the lowest value found in Kyrgyzstan. The largest average home garden size was in Kyrgyzstan (ca. 1755 sqm), compared to the average size for the whole region of 1490 sqm. Consequently, tree density was lower in Kyrgyzstan than in the other two countries. Home gardens were established on average 40 to 50 years before the time of data collection, with some older home gardens (>70 years old) found in Uzbekistan and Kyrgyzstan, and some very young ones (e.g. one-year old garden) established in Kyrgyzstan.

**Fig 3:**
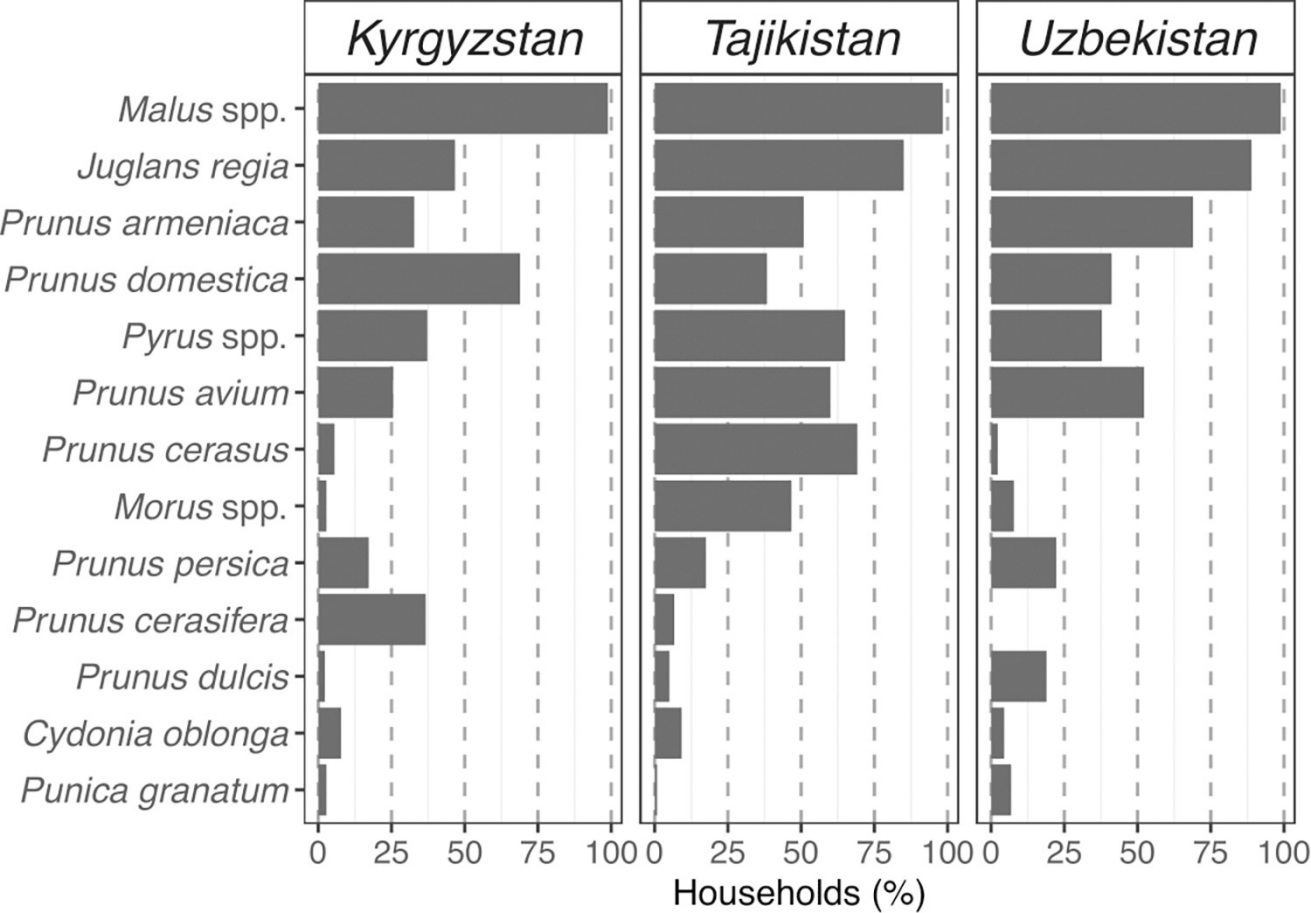
Percentage of households per country growing different tree species in their home gardens.

In Tajikistan and Uzbekistan, most respondents had 5 or 6 plant species in their home garden. The most diverse home gardens were observed in Uzbekistan (11 plant species), but the largest proportion of farmers with 5 to 8 species in their home garden was recorded in Tajikistan. Home gardens in Kyrgyzstan were less diverse, with a mode of 4 species and only a small fraction of respondents having more than 6 species (Fig 2). Apple (*Malus* spp.) was the most common species planted in each country, present in all but five (385/390) home gardens (Fig 3). A large proportion of households (around a third in Kyrgyzstan, and well above in Tajikistan and Uzbekistan) also planted walnut (*Juglans regia*) and apricot (*Prunus armeniaca*) trees. More households were planting these two species in Uzbekistan than in the other two countries. A binomial model applied to test differences between countries indicated that significantly fewer households were planting walnut in Kyrgyzstan, where walnut is harvested mainly in the forest (p = <0.0001). For apricot, differences were significant among all countries (p<0.05) (Fig 3).

Varietal (within-species) diversity was found to be particularly high for five tree species: apple (*Malus* spp.*)*, walnut (*Juglans regia*), apricot (*Prunus armeniaca*), pear (*Pyrus* spp.), and plum (*Prunus domestica*) (Table 4). The largest varietal diversity was reported for apple trees, with a total of 49 varieties named across the three countries, plus additional local varieties to which the farmers could not attribute a name.

**Table 4:** Total and average number of varieties per home garden for five highly represented tree species.

	Kyrgyzstan (no. = 178)	Tajikistan (no. = 118)	Uzbekistan (no. = 89)	Region (no. = 385)
Tot no. apple varieties	42	13	18	64
Average no. apple var./home garden	2.92	2.47	2.33	2.65
Tot no. apricot varieties	12	5	1	15
Average no. apricot var./home garden	0.4	0.55	0.69	0.51
Tot no. walnut varieties	4	4	4	8
Average no. walnut var./home garden	0.47	1.05	0.92	0.75
Tot no. pear varieties	14	6	3	22
Average no. pear var./home garden	0.46	0.92	0.36	0.58
Tot no. plum varieties	10	3	1	12
Average no. plum var./home garden	0.75	0.38	0.39	0.56
Tot no. tree varieties for all tree species in home gardens	97	42	36	132
Average proportion of exotic vs total no. of apple varieties	86%	41%	67%	68%
Average proportion of exotic vs total no. of pear varieties	59%	8%	53%	35%

Kyrgyzstan showed the largest absolute varietal diversity for all species examined except for walnut. Yet, as the number of households sampled differed across countries, the average number of varieties per home garden better accounts for inter-country differences. Using this measure, varietal diversity for apple and plum was highest in Kyrgyzstan, whereas for apricot it was highest in Uzbekistan, and for walnut and pear in Tajikistan. For all countries, walnut (harvested mainly in the wild, especially in Kyrgyzstan) presented the lowest number of varieties (8) among the highly occurring species examined.

**Fig 4:**
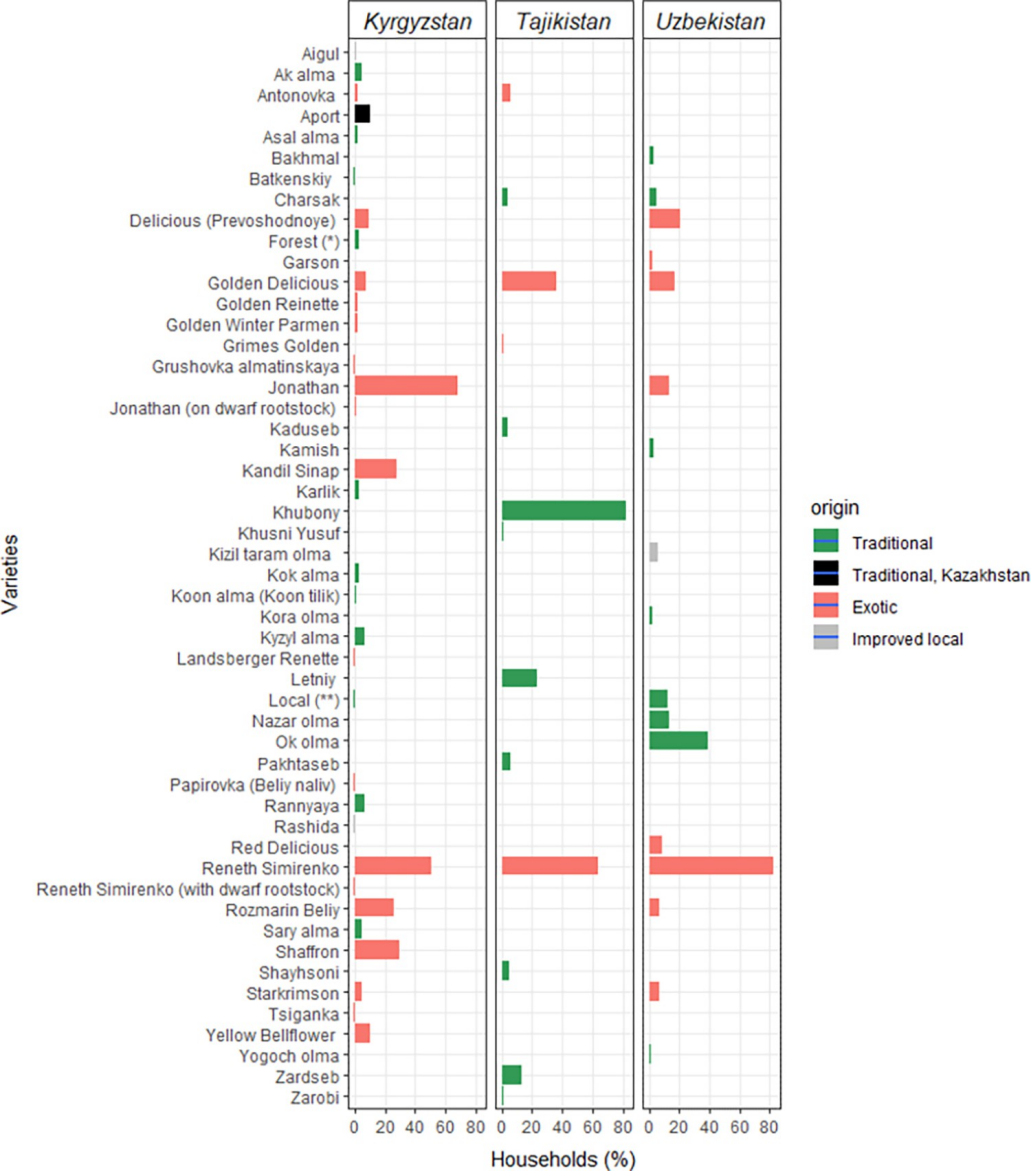
Percentage of households with each apple (*Malus* spp.) variety in their home garden, per country. Forest (*): material derived from seeds/root suckers harvested in the wild (forest). Local (**): local varieties to which the farmers interviewed could not attribute a name. Traditional: varieties available locally and identified through a specific name. Traditional Kazakhstan: traditional varieties that originated from Kazakhstan. Exotic: varieties coming from outside the country, usually widely commercialized. Improved local: varieties that originated within the country and have undergone formal breeding.

‘Reneth Simirenko’ is a common exotic apple variety, present across the three countries and occurring across a large number of households. More than half of respondents in Kyrgyzstan and Uzbekistan, and over 80% in Uzbekistan, reported growing this variety (Fig 4). ‘Golden Delicious’ was also reported in all three countries but present in a lower proportion of home gardens.

A few commercial, exotic varieties were found in common between home gardens in Kyrgyzstan and Uzbekistan: Rozmarin Beliy, Delicious (Prevoshodnoye), and Jonathan. The most common apple variety in Kyrgyzstan was ‘Jonathan’ (grown in ca. 70% of home gardens), introduced from North America, whereas ‘Khubony’, a local landrace, was the most widely grown in Tajikistan, with over 80% of respondents reporting its cultivation. Only one fifth of the varieties were named by more than 20% of respondents. Prevalent varieties of apricot (*Prunus armeniaca*), plum (*Prunus domestica*), and pear (*Pyrus* spp.) differed considerably between countries (S1–S4 Figs). For walnut, only one traditional variety (‘Kogati’) was found in common between Tajikistan and Uzbekistan, while for pear, only one commercial, exotic variety (‘Clapp’s Favorite’) was found in common between the same two countries (S2 and S4 Figs).

Home gardens presented a large share of exotic varieties of apple (in 43% of home gardens with apple, all varieties were exotic) and pear (in 31% of home gardens with pear, all varieties were exotic), whereas traditional varieties of apricot, walnut and plum were most prevalent–particularly local domesticated varieties, to which respondents could not attribute a name. Interviewees referred to some varieties of walnut and plum as ‘*forest*’ varieties, indicating that they sourced their seedlings from local forests (see S2 and S4 Figs).

**Fig 5:**
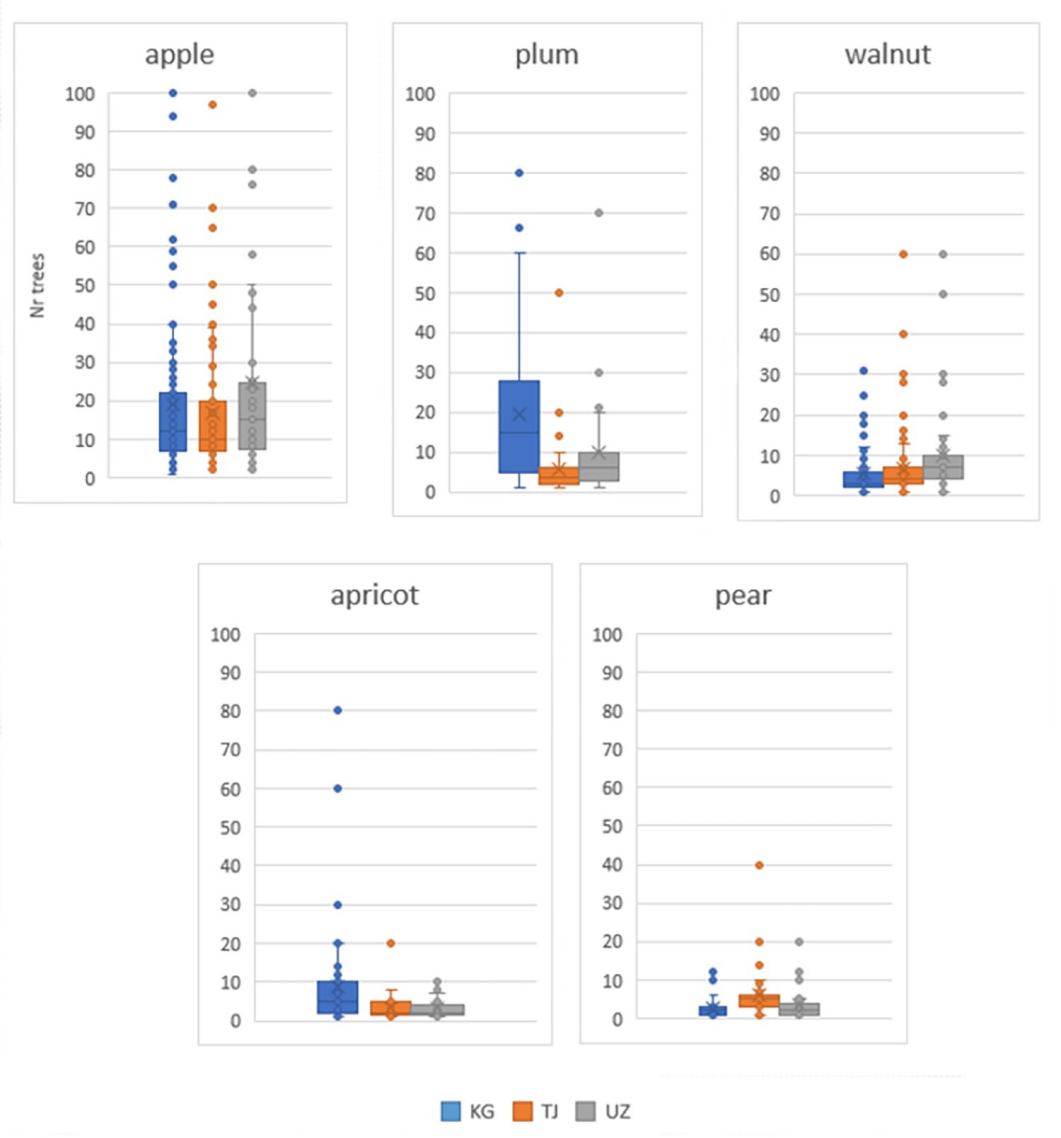
Boxplot with number of individuals for each of the top five most abundant tree species in home gardens of each country. Average values are calculated considering only those home gardens where each species occurred.

**Fig 6:**
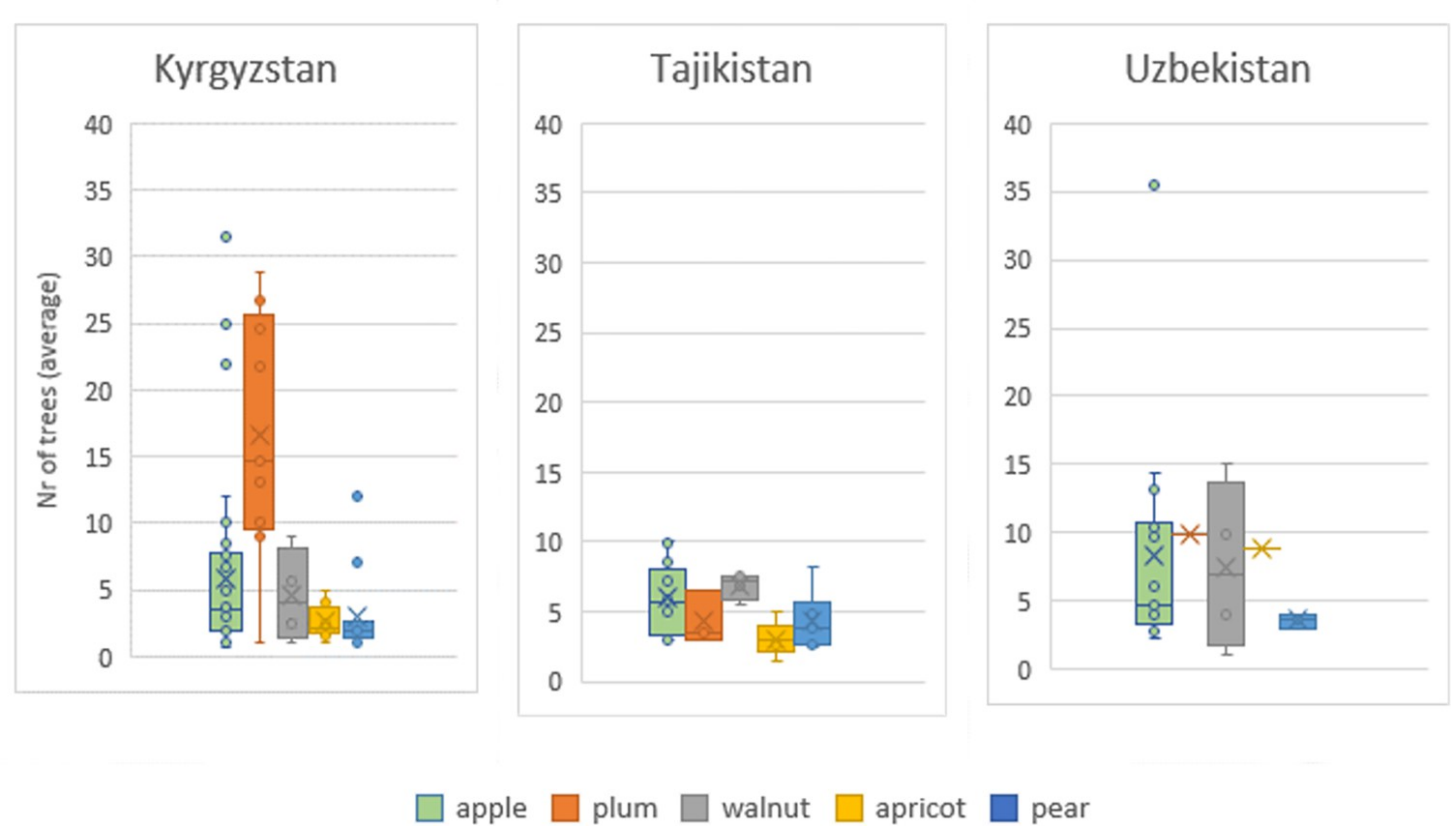
Boxplot with average number of trees per variety, by species and country, for the five most abundant species. Average values are calculated considering only home gardens where each variety occurred.

On average, the highest number of individuals per species was cited for apple, followed by plum and walnut (Fig 5). The largest numbers of trees found per home garden were for apple and walnut in Uzbek home gardens, for plums in Kyrgyz ones, and for apple in Tajik ones. On average, Kyrgyz interviewees reported a larger number of varieties of plum than of other species, and more plum varieties than other countries, whereas Tajik and Uzbek respondents had the largest numbers of varieties of apple and walnut. Some apple varieties with the largest populations (> 20 individuals per home garden) appear as outliers, particularly in Uzbekistan and Kyrgyzstan (Fig 6).

Interviewees explained that most of the species and varieties were consumed at home, and a smaller proportion was sold (see S1 Table). For all varieties of all species, the majority of households kept a greater part of the production for home consumption than for sale. To a much lesser extent, fruit and nuts were also offered as gifts, except in Tajikistan, where this did not seem to be customary. Only in the case of very few varieties did more than 20% of the households sell their production. In all countries, a third or more of households sold the mostpopular appled variety, Reneth Simirenko. A few additional varieties were sold by a significant number of households: Golden Delicious and Khubony in Tajikistan, and Jonathan in Kyrgyzstan. Regarding apricot, one or more varieties falling in the category local were sold by a good share of households (ca. 24%) in Uzbekistan only. For walnut, only in Tajikistan did a good share of households sell their produce for the traditional variety (Kogati) in addition to one or more local variety. Production of pear and plum was mostly reserved for home consumption and, to a much smaller extent, for gifts. The most highly cited characteristics influencing decisions to plant certain varieties were taste (72% of cases), market requirements (43%) and resistance to environmental stresses (19%).

### Sources of planting material

A total of 10 different sources of planting material were reported. According to interviewees, the largest source of planting material in Kyrgyzstan and Tajikistan was the market followed by the forest, whereas in Uzbekistan it was neighbors and friends followed by self-grown planting material maintained through grafting or seed saving (Table 5).

**Table 5:** Sources of planting material by country for all species planted in home gardens.

Source	Freq All %	Freq KG %	Freq TJ %	Freq UZ %	N_All	N_KG	N_TJ	N_UZ
Market	71.47	**73.18**	**90.00**	38.16	375	179	120	76
Forest	36.36	**38.88**	**45.83**	11.11	363	180	120	63
Nurseries	21.98	22.78	15.83	31.25	364	180	120	64
Neighbors, friends	17.37	11.80	5.83	**46.34**	380	178	120	82
Self-grown	14.75	10.56	5.83	**42.42**	366	180	120	66
Forestry Enterprise	8.49	9.44	8.33	6.15	365	180	120	65
Relatives	2.22	0.56	1.67	8.20	361	180	120	61
Projects	1.03	0.56	2.50	0.00	390	180	120	90
Fairs	0.77	0.00	2.50	0.00	389	180	120	89
Research Institute	0.77	0.00	2.50	0.00	390	180	120	90

Note: Frequencies represent the proportion (%) of respondents per country who cited the given source of planting material. Open ended question and multiple responses (sources) per interviewee possible, hence frequencies add up to more than 100% per country. Total number of respondents reported in the last four columns. KG: Kyrgyzstan, TJ: Tajikistan, UZ: Uzbekistan.

Uzbek respondents explained that obtaining planting material from neighbors, friends, or using self-grown material was desirable because it allowed them to know the properties that the planted trees would have. In Uzbekistan, farmers who did not collect planting material from the forest and chose other primary sources believed that collecting from the forest for free was forbidden, and that buying high quality tree grafts from the Forestry Enterprise nursery or local varieties at the local market was possible. In Tajikistan, several farmers stated that the material purchased from the market was of the highest quality (e.g., high value varieties, disease-free), whereas they considered that seedlings from the forest were not grafted so would take a long time to start yielding fruit.

Those who collected planting material in the forest believed that this material was well adapted to local conditions and free of charge. They also noted that some species/varieties of interest to them were not available in the market. In Kyrgyzstan, farmers who did not collect planting material from the forest preferred other sources for various reasons: they did not know how to graft trees, the forest was too far away, or they did not have access to the forest plots allotted to households as long-term leases as part of forest co-management arrangements with the state. Those who collected planting material from the forest appreciated wild seedlings for their potential good root system, which made them ideal as rootstock for grafting high quality trees, and for their reportedly high survival rate. In addition, they stated that wild seedlings did not suffer from root diseases, bore fruits constantly, and needed relatively little water.

Nurseries other than those run by the Forestry Enterprise were managed by smallholder farmers. Often based on specific farmer requests, these small nurseries provided fruit tree saplings grafted with cultivars, which represent a greater varietal diversity than the planting material distributed by the Forestry Enterprise. The market provided all types of planting materials, both from private sources and nurseries run by the government, while nurseries of Forestry Enterprises produced only formal varieties.

A total of 20 criteria were provided to explain the characteristics farmers considered when deciding to plant certain trees from the forest or other sources. The criteria cited by more than 5% of farmers across countries are provided in Table 6. Among these, taste of the future fruit or nut, followed by market requirements for fruit or nut were by far the two most cited characteristics. To be marketable, the products needed to have very good appearance, good taste, and had to meet consumer preferences. From the third position onward, differences emerged by country. Resistance to environmental stresses and adaptation to climate change were of concern to respondents in Uzbekistan, while storability of the fruit was highly cited in Kyrgyzstan, and productivity in Tajikistan.

**Table 6:** Preferred characteristics when sourcing planting material for home gardens, by country.

Characteristics	All Freq (%)	KG Freq (%) (no. = 180)	TJ Freq (%) (no. = 120)	UZ Freq (%) (no. = 90)
Taste	71.79	**73.33**	**71.67**	**68.89**
Market requirements	42.82	**28.89**	**63.33**	**43.33**
Resistance to environmental stresses	19.49	12.78	23.33	**27.78**
Productivity	14.10	9.44	**25.00**	8.89
Storability	13.59	**22.78**	5.00	6.67
Adaptation to local climate	11.28	3.33	10.00	28.89
Known variety of high quality	10.26	11.67	15.83	0.00
Resistance to pests and diseases	5.38	1.67	12.50	3.33

Note: Frequencies are proportions of respondents per country who cited a given characteristic. Multiple responses were possible, hence frequencies add up to more than 100% per country. KG: Kyrgyzstan, TJ: Tajikistan, UZ: Uzbekistan.

As a source of germplasm, the forest provided different types of planting materials: seed, seedlings, rootstocks, and grafts. Seedlings were the most frequently collected material from the forest in Kyrgyzstan and Tajikistan, whereas seeds were more frequently collected in Uzbekistan (Table 7). The traits most frequently cited for influencing the selection of seedlings and of individuals from which to collect seed in the forest were height of the seedlings (generally between 1 and 2 meters) and stem diameter, due to the implications this diameter holds for grafting (Table 8). Respondents also noted that a good root system is critical, as it ensures smooth transplanting, and that age is relevant, as younger seedlings are easier to dig up in the forest.

**Table 7:** Types of planting materials for home gardens sourced from the forest.

Type of planting material (% respondents)	All countries (44%)	KG (50.5%)	TJ (56%)	UZ (14%)
Seedlings	85.38	89.01	89.55	38.46
Seed	19.30	15.38	14.93	69.23
Rootstock	9.94	5.49	17.91	0.00
Graft	0.58	1.10	0.00	0.00

Multiple answers could be provided by respondents. KG: Kyrgyzstan, TJ: Tajikistan, UZ: Uzbekistan.

**Table 8:** Traits influencing the selection of planting material from the forest.

Preferred traits	No. citations
Height (generally 1–2 m)	48
Stem diameter	45
Shape (straight form)	21
Color	21
Overall quality, appearance	20
Good conditions of roots	20
Age (generally 1–3 years)	17
Health conditions	14
Species of interest	11

Traits cited > 10 times by respondents were considered.

About half of respondents who collected planting material from the forest did not follow particular criteria to select a site for collection. In approximately 60% of cases, those who did mainly collected: only in the plots assigned to them under forest lease agreements, in sites managed by the Forestry Enterprise, or in locations close to home or the village where they were entitled to collect planting material and knew they could find good seedlings. In the remaining 40% of cases, farmers reported primarily targeting sites with abundant seedlings of species of interest (eg., pear, apple, walnut) of good quality. Reported survival rates of planting material sourced from the forest were high (>75%) in all three countries.

### Planting trees in the forest

More than half of the farmers in Kyrgyzstan (58%) and Tajikistan (52%) planted trees into the forest, whereas significantly fewer farmers planted trees in the forest in Uzbekistan (25%) (p<0.001). The main reason reported for planting trees in the forests was a perceived obligation to do so by the Forestry Enterprise (Table 9). Other reasons included: the objective to promote the establishment and expansion of the forest, increase the density of species of interest, and obtain valuable products for one’s household and for future generations. Several tree species were planted into the forest: from 15 in Tajikistan to 8 in Uzbekistan. These include *Ulmus* spp., *Robinia pseudoacacia*, *Populus* spp., *Juniper* spp., and *Pinus* spp., which were not reported as home garden species. Walnut is predominantly planted in the forest in all three countries (particularly in Kyrgyzstan), followed by apple (Table 10). Reported survival rates were > 50%.

**Table 9:** Top-cited reasons for planting trees in the forest, all countries examined jointly.

Reasons	No. citations
Requirement by the Forestry Enterprise	39
Promote forest establishment and development; expand forest area and improve leased forest plots	22
Increase density of trees (especially of species of high interest, primarily walnut, but also cherry plum/alycha, and other fruit tree species)	19
Obtain edible products and construction material for direct use and income generation	19
Support conservation	13
Fill gaps in the forest	11
Beneficial task	11
Professional duty; past work experience in Forestry Enterprise	11
Protection, prevention of landslides, reduction of erosion, bank stabilization	10

**Table 10:** Percentage of households per country and overall that plant trees into the forest for each species planted.

Scientific name	Common name	All %	KG % (no. = 180)	TJ % (no. = 120)	UZ % (no. = 90)
*Juglans regia*	walnut	33.1	42.2	30.8	17.8
*Malus* spp.	apple	19.0	21.7	22.5	8.9
*Prunus cerasifera*	cherry plum	6.7	10.0	6.7	0
*Prunus domestica*	plum	5.4	4.4	9.2	2.2
*Prunus cerasus*	cherry	4.6	0	14.2	1.1
*Ulmus* spp.	elm	3.3	6.7	0.9	0
*Robinia pseudoacacia*	acacia	3.3	1.1	9.2	0
*Prunus dulcis*	almond	2.8	0.6	6.7	2.2
*Populus* spp.	poplar	2.6	2.2	5.0	0
*Prunus armeniaca*	apricot	1.8	1.7	0.8	3.3
*Pyrus* spp.	pear	1.5	0.6	4.2	0
*Morus* spp.	mulberry	1.0	0	3.3	0
*Juniper* spp.	juniper	0.5	0	1.7	0
*Prunus avium*	sweet cherry	0.5	0	1.7	0
*Cydonia oblonga*	quince	0.5	0	1.7	0
*Prunus persica*	peach	0.3	0	0	1.1
*Pinus* spp.	pine	0.3	0	0	1.1
Tot no. species			10	15	8

For some plants, respondents gave the common name and the genus was subsequently identified.

The main sources of reproductive material planted in the forest were from the Forestry Enterprises (Table 11). These have their own nurseries where seed harvested from the forest is used to produce seedlings for forest restoration purposes.

**Table 11:** Source of reproductive material planted in the forest.

Source (% respondents)	All (52.6%)	Kyrgyzstan (60.6%)	Tajikistan (59.2%)	Uzbekistan (27.8%)
Forestry Enterprise	28.2	31.67	35.83	11.11
Home garden	7.69	7.78	8.33	6.67
Nurseries	4.87	3.33	5	7.78
Forest	4.36	9.44	0	0
Neighbors	3.59	2.22	8.33	0
Protected woodland	1.54	3.33	0	0
Leased land	1.28	2.78	0	0
Market	0.51	0	0.83	1.11
Relatives	0.51	0	0.83	1.11

Percentages are calculated on the total survey participants who plant in the forest.

### Motivations to maintain home gardens

The large majority (95%) of respondents indicated that they wanted to continue cultivating fruit and nut trees in their home gardens in the future. Their primary motivation was that home gardens constitute a main livelihood resource, bringing income and healthy edible products to support balanced diets. Others indicated that continuing fruit and nut tree cultivation is a way to maintain capital inherited or passed down to them by their predecessors, who established the home garden. Others still emphasized the value that these home gardens hold for future generations (children and grandchildren). Finally, some respondents stated that to be settled in the village and manage a home garden is a way of living that they like and wish to maintain as long as their health allows.

A large percentage (82%) of interviewees across the three countries intended to expand their tree planting. Those who did not are constrained by a lack of land and only intended to replace old trees. The main motivation for planting more trees was to take advantage of empty land to increase fruit and nut production for sale, improve income, and test new varieties while replacing old trees. Nearly all (94%) respondents were positive/hopeful about their children staying in the village, and that at least one son (usually the youngest) would continue home garden and orchard management.

## Discussion

### Species diversity

According to our findings, home gardens across the three countries comprised similar species. This is unsurprising given that agroecological conditions, traditional farming systems and the recent historical, economic and political trajectories of the countries examined have much in common. The most prevalent species in all three countries were apple (*Malus* spp.), found in almost 100% of home gardens, and walnut (*Juglans regia*), plum (*Prunus domestica*), and apricot (*Prunus armeniaca*) were also common species. Apple, walnut and plum were the only three species that occurred in more than half of the home gardens, for all countries examined jointly.

Population sizes of different species were generally small (on average between 5–10 individuals/species), except for plums in Kyrgyzstan. The largest numbers of trees found per home garden were apple and apricot in Uzbekistan, and plums in Kyrgyzstan, and apple in Tajikistan. Average population sizes of tree species were the largest for apple. On average, approximately 20–30 apple trees were reported in home gardens, with a large spread of values. Other studies have also reported the dominance of apple in home gardens in temperate regions [54] and indicated that apple is the most widespread and well-adapted species of temperate fruit crops, and is the fourth most important world fruit crop after species of the genus Citrus, grapes and bananas.

Across countries, the mean number of tree and shrub species in home gardens was 4.7, with a minimum of 1 and a maximum of 11 species per home garden. Tree species diversity per home garden was slightly higher in Tajikistan, where the highest proportion of home gardens with 5–7 species was found, compared to the other two countries, which had a lower number of species per garden. The least diverse home gardens, with the smallest number of species reported, were in Kyrgyzstan, perhaps reflecting their relatively recent history of switching from pastoralism to settled agriculture [41, 55].

Accounts of species diversity in home gardens in Central Asia are very limited in the English-language scientific literature, but the few accessible research findings align well with the results from this study. Currey [53] investigated diversity in home gardens in Kyrgyzstan and found a similar species composition, with an average of 6.4 species per home garden. Van Dusen [56] indicated an average of 4.9 fruit and nut tree species per home garden in Uzbekistan, similar to our average of 4.8 species per home garden in Uzbekistan in our study. Van Dusen [56] also found that the number of fruit trees varied largely (2–25 trees per garden in central parts of the valley he investigated, and 4–100 in more peripheral parts) depending on access to water. Apple, persimmon, and walnut were preferred by households because they produce later in the year than most other trees and can be preserved during the cold period, when little to no fresh produce is available. Grapevines were appreciated for their fruit and shade.

In a 2009 survey on Kyrgyz home garden diversity [53], households were more likely to report species they sold, and less likely to report non-commercial species. If this also holds true in our study, the diversity reported may be less than the total richness of species and varieties that actually exists in the home gardens studied.

There are potential issues associated with the classification of diversity, especially of wild relatives. Most species in the genus *Malus* (about 27 wild species recorded in the literature) intercross, given their self-incompatibility, and novel diversity is occasionally found and described in wild apple populations [57]. This means that species names are not always certain. However, even if the number of named varieties cannot be directly considered a proxy for genetic variation, these folk variety names are often attributed based on phenological variation at population level, which in some cases reflects genetic differences. There are examples of high levels of agreement between folk variety names and distinct varieties defined based on molecular markers [58].

### Varietal diversity

In our study, each species showed very different patterns with regard to varietal diversity, with very few varieties reported in common across countries. Yet, we cannot exclude the possibility that respondents used different names to refer to the same variety. Varietal diversity was high for walnut, apricot, pear, and plum, but by far, the highest varietal diversity was found in apple, which presented some varieties in common across all three countries. The most widely found was Reneth Simirenko: a very popular variety, formally bred, particularly appreciated on the market, and easy to store, introduced from Russia about a century ago, and well acclimatized to the Central Asian regions. Other varieties were also shared between at least two of the three countries; however, all these common varieties were commercial ones. While for apple and pear close to half of the varieties in home gardens were commercial ones, apricot, walnut and plum varieties were almost exclusively traditional ones. Similarly, so called ‘local’ varieties, which are not formally classified, were predominant for apricot, walnut and plum. It was not possible to determine whether one or more varieties were included by farmers under this definition. ‘Local’ apricot varieties were found in >60% of home gardens in Uzbekistan, the second largest apricot producer globally after Turkey [59]. ‘Local’ walnut varieties were found in >80% of home gardens in Uzbekistan, whereas in Kyrgyzstan, the most prevalent variety was ‘forest’-sourced, and in Tajikistan the dominant variety was ‘Kogati’, also present in Uzbekistan. ‘Local’ plum varieties were found in ca. 30% of home gardens across the three countries (and ca. 40% in Uzbekistan).

Kyrgyzstan showed the largest absolute varietal diversity for all studied species except for walnut. However, when measured by average number of varieties per home garden, varietal diversity is only highest for apple and plum in Kyrgyzstan, while Uzbekistan boasts the highest varietal diversity for apricot, and Tajikistan for walnut and pear.

Although formal institutions develop and release high performing varieties, much of the material used in home gardens is locally developed. Currey [53] indicated that, in Kyrgyzstan, only 10% of households identified apples as ‘wild’ in their home garden. However, almost all rootstocks of ‘cultivated’ apple and apricot varieties in home gardens were from locally sourced wild apple or apricot, most commonly derived from seed collected in the valleys surrounding the villages. Cultivated varieties were then grafted on these wild rootstocks. Wild apples were not classified by home gardeners as separate species, but as an apple variety.

### Maintenance and/or loss of biodiversity

The findings from this study are in line with those from a regional UNEP-GEF project (2006–2012) that found a large diversity of local fruit trees in the region, despite the loss of many local varieties (see additional information in the online project portal [60]). Researchers in that initiative found that some species and varieties had very small population sizes and were sometimes represented by one single individual (Turdieva M., *personal observation*). Diversity loss in home gardens is ongoing in Central Asia [61] and other regions, due to the extinction of ancient varieties of local fruit trees generally replaced by commercial varieties [62], and the conversion of land to other uses, resulting in the loss of fruit tree wild relatives’ habitats [63, 64]. Thus, despite our results showing that home gardens in Central Asia are quite diverse systems, there are concerns about future evolutions.

Van Dusen [56] observed that the way crop genetic resources are maintained in home gardens differs considerably from perennial tree crops. Although few tree individuals are kept in each home garden, their overall intraspecific diversity may be larger than for annual crops. Most fruit trees and grapes are clonally propagated, such that farmers are not managing populations but are constantly replacing or renewing genetic lines to overcome the lack of long-term stability of small populations. Particularly interesting is the case of apple genetic resources. The domesticated apple appears to be resulting from an evolutionary process that took place over thousands of years. The species *Malus sieversii*, that spread over Central Asia and in the Tian Shan Mountains, has been identified as the main contributor to the gene pool of the cultivated apple (*Malus domestica*), and its hybridizations with other wild apple species along the Silk Route have generated the diversity currently present in the domesticated apple [65].

Interestingly, despite the large traditional use of clonal propagation by grafting of elite cultivars, studies of cultivated apples do not reveal domestication bottlenecks. This has been attributed to different drivers that have maintained large genetic variation: several farmers autonomously selected trees producing fruits with desirable traits, from progeny deriving from natural pollination, the obligate outcrossing of the species, the isolation of farms, and the geographic variation in taste preferences that have led to different selection criteria [66].

However, despite its high genetic variability manifest across the thousands of cultivars distributed throughout the world, the size of apple genetic resources used by breeders has been limited to a few highly related cultivars that account for a large share of the global production (particularly four of them: Golden Delicious, Gala, Red Delicious, and Idared) [65]. Consequently, many interesting and well adapted traditional and local varieties have been abandoned and have been partly lost [61]. Furthermore, the genetic resources of the wild relative *M*. *sieversii* are increasingly threatened as populations experienced severe overexploitation during the Soviet period, the remaining populations are under pressure by forest destruction, and wild apple species show a high degree of introgression from domesticated apple, which may undermine their genetic integrity [67].

### Choices around diversity in home gardens

Well recognized motivations for farmers to maintain diversity, especially of traditional varieties, are: their adaptation to the local context (e.g., pest resistance, capacity to grow on marginal lands, temperatures and soil conditions, presence or lack of irrigation), association with foods that are connected to cultural identity, diversity in traits of interest, reflecting a diverse genetic composition, and access to a processing industry and markets for the variety [54, 61]. In our study, the main criteria reported to decide which species and varieties to plant in home gardens were aligned across the three countries. Overall, taste was the most important characteristic, followed by market requirements. This suggests a significant market orientation, even though most of the home garden production was reportedly for home consumption, with only a surplus fraction destined for sale (particularly for highly commercial varieties). Differences in preferred varietal attributes across countries included a higher emphasis on environmental stresses and adaptation to climate change in Uzbekistan, storability in Kyrgyzstan, and productivity in Tajikistan. Criteria related to cultural aspects were not explicitly reported.

Even if home gardens were originally established to supply food for the household, new market opportunities may increasingly influence the choice of diversity to be cultivated (see also [68]). The above-mentioned UNEP-GEF project (2006–2012) shows that while home gardens still include traditional varieties for self-consumption and local markets, large orchards exclusively grow commercial varieties for local and export markets (Turdieva M., *personal observation*). Commercialization, market forces, youth migration [69, 70], and generational changes can pose a threat to home garden diversity. In fact, various studies highlight that the increased specialization and commercialization of agricultural production often leads to a loss of diversity in cropping systems [71, 72], given the strong influence of economic returns on the choice of species cultivated [73, 74]. However, according to Currey [53] and the authors he cites [41, 55], Kyrgyz home gardens were not transitioning from subsistence to commercialization, as observed in other contexts. Rather, these have fulfilled both food production and sales functions from the beginning and continue to do so. These authors call for more research on the link between market pressures and cultivated plant diversity [41, 53, 55].

A recent study found that walnut genetic diversity in home gardens of Kyrgyzstan, Tajikistan and Uzbekistan was comparable to that of walnut tree populations found in nearby forests [75]. This suggests that home gardens, at least for some species largely represented by traditional, local varieties, could still encompass significant levels of diversity, similar to what could be found in the ‘wild’. In Tajikistan, where fruits are a main source of food and income for rural households, authors [68] distinguish between many local varieties maintained for household consumption and others introduced for income generation, and note that a pure market focus could endanger household food and nutrition security.

The diversity found in home gardens reflects the greater dynamism that has characterized home gardens compared to other agricultural land in the Central Asia region, given that farmers had more autonomy to decide what to cultivate in these small garden plots managed at household level. In Uzbekistan, Van Dusen [56] indicates that among all forms of tenure, the horticultural sector associated with home gardens has traditionally been the most independent from governmental control, and thus more able to respond to new market opportunities. As opposed to growing cotton or wheat, which are influenced by production quotas, fixed pricing, centralized management of administrators of collective and state farms, revenue from growing fruit enables households to directly access cash-based markets. Van Dusen [56] further considers that greater autonomy in farmers’ decision-making in relation to home gardens helps explain the diversity found in these systems.

### Sourcing of planting material

Planting material used for home gardens comes from various sources. Across the three study countries, genetic material was formerly distributed by collective farms in Soviet times, but that role has been progressively taken up by regional and district markets, with a concomitant rise in private sector propagators and traders of genetic material [56]. For instance, a market study [76] shows rising Uzbek imports of fruit tree rootstocks for orchards from Turkey, China, and Italy. Other authors [5] identify more than 100 commercial nurseries involved in growing seedlings of fruit crops and grapes within farm and forestry enterprises in Uzbekistan. In our analysis across the three study countries, planting material was sourced predominantly from the market, followed by the forest in Kyrgyzstan and Tajikistan. Prevalent sources of planting material in Uzbekistan differed from the other two countries: neighbors and friends were first, followed by self-production. A large informal seed network seemed to exist in Uzbekistan, possibly explained by a preference for material adapted to local conditions, tested and recommended by neighbors and friends. The main factors driving the choice of planting material across the three countries were quality, cost, and access (including distance to the material).

Distinct land reform processes across the study countries may explain some of the country-specific patterns choices regarding planting material used in home gardens, described below. Each country of the former Soviet Union designed different land reform and distribution policies, which resulted in the uneven occurrence of collective farms, private farms, household plots, or home gardens. Different levels of implementation of land reforms may explain some of the differences across and within countries in sourcing of planting material. For instance, some authors [77] found that the ambitious agricultural reforms set in place in post-Soviet Russia resulted in a profound restructuring in some regions, while in others, Soviet-style coordination remained. Some farmers thus gained more independence from former structures, while others retained their dependence on subsidized inputs.

Across countries, saplings of both traditional and commercial varieties not formally registered are sourced from the market, while Forestry Enterprises and research institutions primarily distribute saplings of certified commercial improved varieties, both local and exotic [5]. Smallholder private nurseries are the most diverse sources of planting material of local fruit tree varieties, many of which are not included in the State Register of Plant Varieties, a catalog of officially recognized registered varieties [5]. Originally, variety registration developed along with advances in plant breeding with the objective to create transparency in the marketplace. It is also a source of information on the agronomic value of a variety, and supports a seed certification system (see [78]). The type of nursery business ownership is likely to vary significantly among countries. At the time of our study, private nurseries were still limited in Uzbekistan, and there were examples of private forestry-oriented nurseries in Tajikistan, as well as a very active NGO sector indirectly supplying financial resources for tree nurseries in Kyrgyzstan (Judy Loo, *personal observation*).

In a study conducted in Uzbekistan [56], farmers identified the market as an important source of planting material, but they also largely exchanged planting material locally. When attending district-level markets, farmers still approached vendors from their locality of origin. They also used their own planting material and other informal sources, thereby demonstrating the importance of the informal seed system, despite the significant centralization efforts in the agricultural sector during Soviet rule. In Uzbekistan, despite the dominant role of the Schroeder Institute in developing and distributing fruit varieties [79], village social networks have remained an important source of genetic resources [80]. The combination of different formal and informal seed sources (government, market, and social networks) illustrates the existence of a dynamic seed system that supports a flow of diverse plant material in home gardens.

A flow of material was observed from the forest to home gardens and vice versa. Overall, the forest was a source of seedlings, although in Uzbekistan seeds were the main type of reproductive material collected there. Criteria used to select planting material were mainly height and stem diameter, the latter of which is particularly relevant for grafting. Wild material was collected in the forest because it was considered well adapted to local conditions and useful as rootstock for grafting with improved varieties in home gardens. Collection sites were limited and largely determined by farmers’ rights to collect in their leased plots, and by the occurrence of the species of interest. Material sourced from the forest had a high survival rate, probably due to a careful selection of saplings in optimal conditions, and their adaptability to local conditions.

More than 50% of study respondents from Kyrgyzstan and Tajikistan planted trees into the forest, while significantly fewer (25%) farmers did so in Uzbekistan. Planting in the forest was mainly organized by Forestry Enterprises as a form of ecosystem restoration. It was also a way for individuals to increase the number of young and healthy trees in their leased plots, replacing aged and sick trees, in return for using the plots for harvesting non-timber forest products and grazing. Reproductive material for forest planting was largely provided by the Forestry Enterprise and usually taken from its nurseries, grown from seeds collected in the forest (not grafted with cultivars), although home gardens were themselves the second most significant source of planting material overall for the three countries. The diversity of species used for forest restoration varies within each country depending on the diversity of conditions where planting takes place. Walnut-fruit forests are species-rich, multi-layered systems, so a range of species are planted there, including juniper and pines at higher altitude, elm and poplar along rivers, and robinia in very dry sites.

### Future prospects

Interest in maintaining, and even expanding, fruit and nut tree planting was high. Most respondents flagged the benefits of home gardens, which were in many cases their main source of income. Most respondents hoped that the cultivation of fruits and nuts could be maintained, and many were counting on at least one son to continue managing fruit and nut trees on the farm. According to tradition, in Kyrgyzstan, Uzbekistan, and Tajikistan [54, 55], the youngest son inherits his parents’ assets. This practice, with historical origins in the transfer of livestock, now also includes housing, home gardens, and additional land under private ownership.

Despite cultural differences, home gardens were established under similar circumstances in the three countries, where they have retained a very important income-generating role, despite their small size and limited surplus production. Income derived from the sale of home garden products is one of the only ways to obtain cash in a context where little cash is exchanged.

Further inventories and research are needed to capture the temporal dynamics of home gardens. For example, are young couples establishing new home gardens as they form their own households? If all sons move away, are local varieties gradually replaced by new commercial ones, and are some local varieties lost when gardens fall into disuse? Genetic erosion of cultivated biodiversity has been reported as an important threat in the region, due to severe pressure on wild stands, which are affected by deforestation and overexploitation. The extinction of local varieties has been recorded in other studies conducted in the region [61], mainly as a result of the introduction of uniform high-yielding varieties, fertilizers and pesticides, and agricultural mechanization, which have supported the expansion of monocrop agriculture. The loss of local varieties also owes partly to the lack of awareness about their value and properties, and to the ecological degradation of wild sources of diversity, associated with anthropogenic activities such as grazing and unsustainable harvesting of forests. Inadequate coordination among conservation institutions, farmers, scientific institutes, government agencies, and the private sector, as well as outdated technologies used to document wild resources, prevent the adoption of an effective, integrated approach [61].

## Conclusions

Although the diversity of species and varieties recorded in home gardens in our study are considerable, there are risks for the long-term resilience of these systems due to the small population sizes of individual species and varieties, the pressures on natural stands and habitats where valuable tree species are found, and the progressive orientation of farmers’ choices towards commercial varieties. Although home gardens primarily sustain household consumption, farmers are favoring plant varieties that are easily saleable on the market to be able to liquidate their surplus production.

Home gardens continue to play a critical role in rural livelihoods in the study countries and are likely to maintain their importance, as they may suitably grow on marginal areas, in a region where most arable land is already being exploited. More targeted research is needed to enhance the contributions of home gardens to food and nutrition security and agrobiodiversity conservation. Linking home garden conservation efforts with national programs would enable the integration of home gardens in the national research and extension system, to better monitor their diversity over time. This study provides a baseline for future studies to understand the trajectories of these critical yet understudied systems.

## Supporting information

S1 Fig Percentage of households with apricot (*Prunus armeniaca*) varieties in their home gardens, for each country separately. Forest (*): material derived from seeds/root suckers harvested in the wild (forest). Local (**): local varieties to which the farmers interviewed could not attribute a name. Traditional (varieties available locally and identified through a specific name). Improved local (varieties that originated within the country and have undergone formal breeding).
(PDF)

S2 Fig Percentage of households with walnut (*Juglans regia*) varieties in their home gardens, for each country separately. Forest (*): material derived from seeds/root suckers harvested in the wild (forest). Local (**): local varieties to which the farmers interviewed could not attribute a name. Traditional (varieties available locally and identified through a specific name). Improved local (varieties that originated within the country and have undergone formal breeding).
(PDF)

S3 Fig Percentage of households with pear (*Pyrus* spp.) varieties in their home gardens, for each country separately. Local (**): local varieties to which the farmers interviewed could not attribute a name. Traditional (varieties available locally and identified through a specific name). Exotic (varieties coming from outside the country, widely commercialized). Improved Kazakhstan (varieties that originated from Kazakhstan and has undergone formal breeding).
(PDF)

S4 Fig Percentage of households with plum (*Prunus domestica*) varieties in their home gardens, for each country separately. Forest (*): material derived from seeds/root suckers harvested in the wild (forest); Local (**): local varieties to which the farmers interviewed could not attribute a name; Sortovoy (***): unknown cultivated variety: improved varieties developed by the national formal breeding program and exotic varieties to which the farmers interviewed could not attribute a name. Traditional (varieties available locally and identified through a specific name). Exotic (varieties coming from outside the country, widely commercialized). Improved local (varieties that originated within the country and have undergone formal breeding).
(PDF)

S1 Table Main apple varieties of highly represented species in home gardens, and their uses by country. Varieties that were used by less than 10% of farmers in all countries were excluded from this table. The values in the column with frequencies refer to the total of respondents by country, not just the subset that claimed to have the species. In the same way the other columns are percentages referring to the total of the sample, not the fraction of people who indicated that they have the species or variety. In this way the frequencies are comparable at least within the single country. When the value of the columns home / sale / etc. coincides with Freq it means that all the owners of that variety use it for that purpose. Totals calculated across uses (home consumption, sale or gift) for a particular variety may exceed 100% since households use the same variety for more than one purpose. Forest (*): material derived from seeds/root suckers harvested in the wild (forest); Local (**): local varieties to which the farmers interviewed could not attribute a name; Sortovoy (***): unknown cultivated variety: improved varieties developed by the national formal breeding program and exotic varieties to which the farmers interviewed could not attribute a name.
(PDF)

S1 Questionnaire (DOCX)

S2 Questionnaire (DOCX)
