# Supplementary material for: Home gardens of Central Asia: Reservoirs of diversity of fruit and nut tree species
Source: PLoS One. 2022 Jul 28;17(7):e0271398. doi: 10.1371/journal.pone.0271398 (PMC9333230; doi:10.1371/journal.pone.0271398)
Supplement: S2 Fig — Forest (*): material derived from seeds/root suckers harvested in the wild (forest). Local (**): local varieties to which the farmers interviewed could not attribute a name. Traditional (varieties available locally and identified through a specific name). Improved local (varieties that originated within the country and have undergone formal breeding). (PDF) [file pone.0271398.s002.pdf]

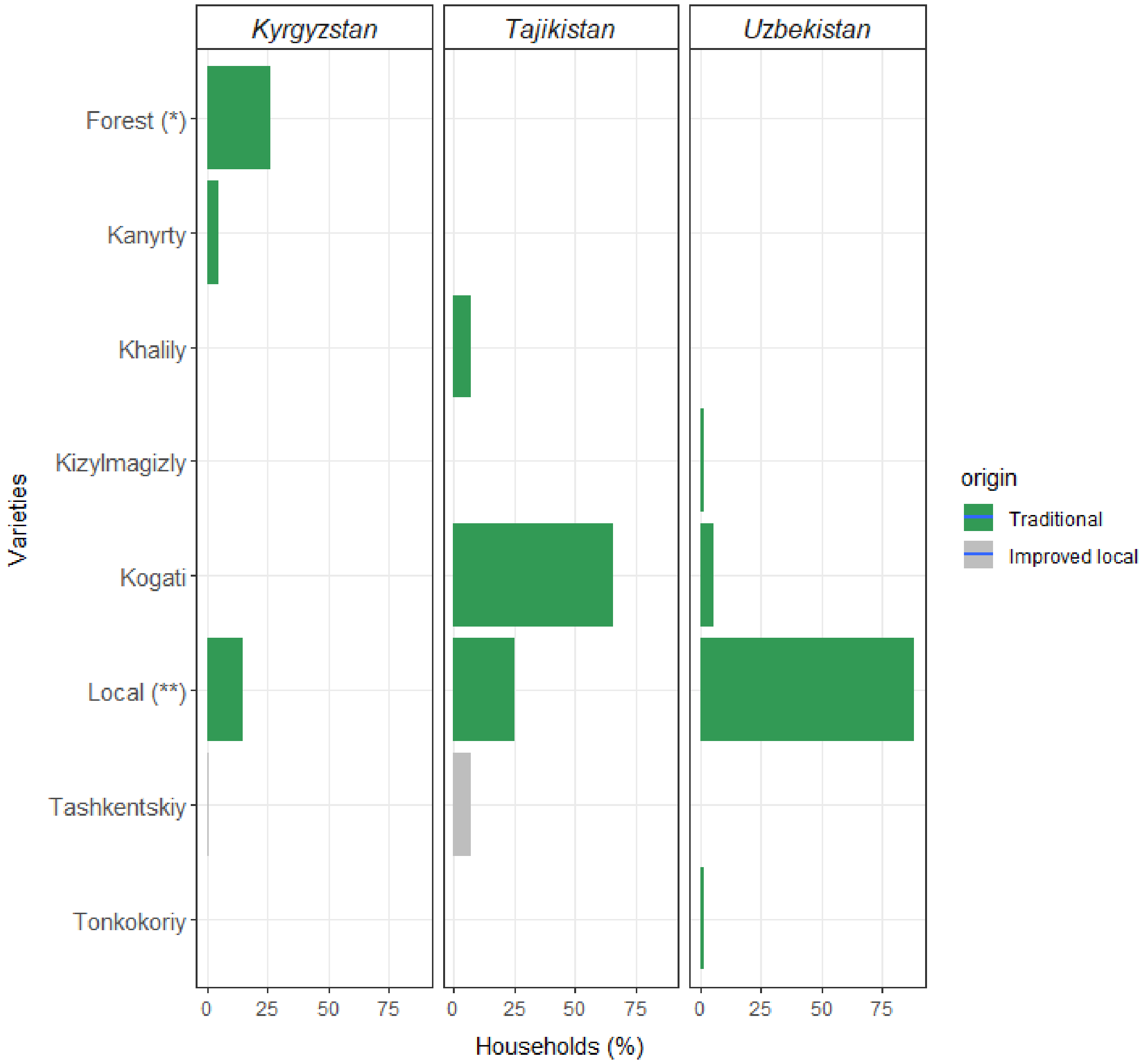

S2 Fig. Percentage of households with walnut (*Juglans regia*) varieties in their home gardens, for each country separately. Forest (*): material derived from seeds/root suckers harvested in wild (forest). Local (**): traditional local varieties to which the farmers interviewed could not attribute a name.
