# Supplementary material for: Home gardens of Central Asia: Reservoirs of diversity of fruit and nut tree species
Source: PLoS One. 2022 Jul 28;17(7):e0271398. doi: 10.1371/journal.pone.0271398 (PMC9333230; doi:10.1371/journal.pone.0271398)
Supplement: S3 Fig — Local (**): local varieties to which the farmers interviewed could not attribute a name. Traditional (varieties available locally and identified through a specific name). Exotic (varieties coming from outside the country, widely commercialized). Improved Kazakhstan (varieties that originated from Kazakhstan and has undergone formal breeding). (PDF) [file pone.0271398.s003.pdf]

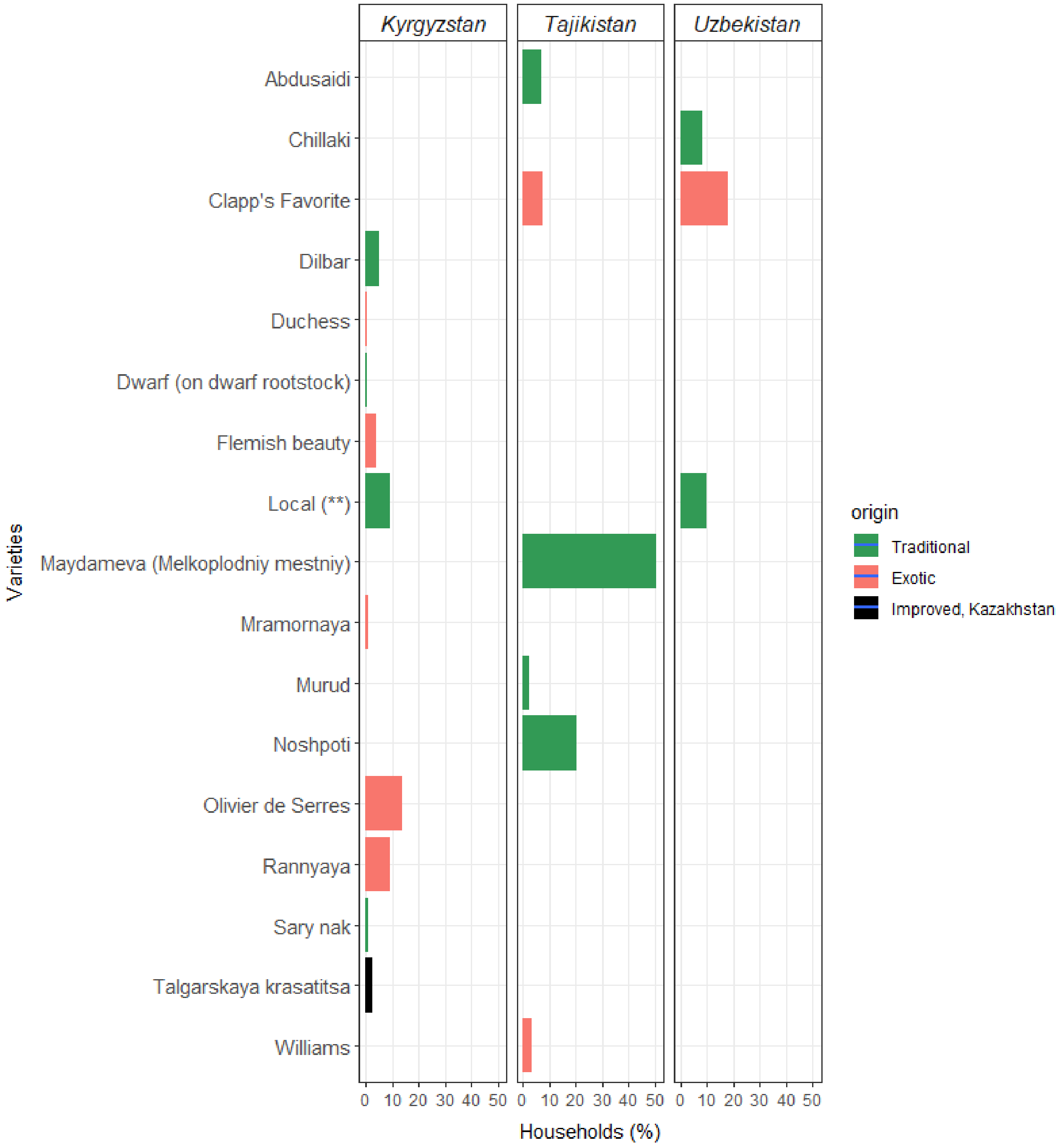

S3 Fig. Percentage of households with pear (*Pyrus* spp.) varieties in their home gardens, for each country separately. Local (**): traditional local varieties to which the farmers interviewed could not attribute a name.
