# Supplementary material for: Home gardens of Central Asia: Reservoirs of diversity of fruit and nut tree species
Source: PLoS One. 2022 Jul 28;17(7):e0271398. doi: 10.1371/journal.pone.0271398 (PMC9333230; doi:10.1371/journal.pone.0271398)
Supplement: S1 Table — Varieties that were used by less than 10% of farmers in all countries were excluded from this table. The values in the column with frequencies refer to the total of respondents by country, not just the subset that claimed to have the species. In the same way the other columns are percentages referring to the total of the sample, not the fraction of people who indicated that they have the species or variety. In this way the frequencies are comparable at least within the single country. When the value of the columns home / sale / etc. coincides with Freq it means that all the owners of that variety use it for that purpose. Totals calculated across uses (home consumption, sale or gift) for a particular variety may exceed 100% since households use the same variety for more than one purpose. Forest (*): material derived from seeds/root suckers harvested in the wild (forest); Local (**): local varieties to which the farmers interviewed could not attribute a name; Sortovoy (***): unknown cultivated variety: improved varieties developed by the national formal breeding program and exotic varieties to which the farmers interviewed could not attribute a name. (PDF) [file pone.0271398.s005.pdf]

Species	Uzbekistan						Tajikistan						Kyrgyzstan					
	Malus spp. (apple)																	
Varieties	Freq. (%)	home	sale	gift	exchange	other	Freq. (%)	home	sale	gift	exchange	other	Freq.(%)	home	sale	gift	exchange	other
Reneth Simirenko	80.49	61.11	32.22	11.11	0	0	64.17	64.17	45.83	0	0	0	50.56	48.33	29.44	4.44	0	0
Ok olma	38.64	35.56	10	5.56	0	0	0	0	0	0	0	0	0	0	0	0	0	0
Delicious (Prevoshodnoye)	20	15.56	2.22	5.56	0	0	0	0	0	0	0	0	0	0	0	0	0	0
Golden Delicious	16.67	13.33	8.89	1.11	0	0	37.5	37.5	26.67	0	0	0	3.33	2.78	1.11	0	0	0
Nazar olma	13.33	12.22	5.56	1.11	0	0	0	0	0	0	0	0	0	0	0	0	0	0
Jonathan	13.48	14.44	2.22	3.33	0	0	0	0	0	0	0	0	68.89	68.33	30.56	6.11	0	0
Local (**)	13.33	11.11	6.67	1.11	0	0	0	0	0	0	0	0	0.56	0.56	0	0	0	0
Rozmarin Beliy	6.67	6.67	0	2.22	0	0	0	0	0	0	0	0	26.11	25.56	7.22	0.56	0	0
Kandil Sinap	0	0	0	0	0	0	0	0	0	0	0	0	16.11	15.56	5	0.56	0	0
Zardseb	0	0	0	0	0	0	14.17	12.5	11.67	0	0	0	0	0	0	0	0	0
Khubony	0	0	0	0	0	0	82.5	82.5	56.67	0	0	0	0	0	0	0	0	0
Letniy	0	0	0	0	0	0	25	25	14.17	0	0	0	0	0	0	0	0	0
Shaffron	0	0	0	0	0	0	0	0	0	0	0	0	29.44	28.89	7.22	2.78	0	0
Species	*Prunus armeniaca* (apricot)																	
Local (**)	68.89	64.44	24.44	10	0	1.11	17.5	17.5	6.67	0	0	0	10.56	8.33	1.67	0	0	0.56
Falgari	0	0	0	0	0	0	20.83	20.83	5.83	0	0	0	0	0	0	0	0	0
Kandak	0	0	0	0	0	0	14.29	14.17	3.33	0	0	0	0	0	0	0	0	0
Species	*Juglans regia* (walnut)																	
Local (**)	88.51	78.89	52.22	16.67	0	0	25.83	25.83	20	0	0	0	14.44	11.67	10	0	0	0
Kogati	5.33	3.33	2.22	2.22	0	0	65.83	65.83	51.67	0	0	0	0	0	0	0	0	0
Forest (*)	0	0	0	0	0	0	0	0	0	0	0	0	27.22	20.56	15.56	3.33	0	0
Species	*Pyrus* spp. (pear)																	
Clapp's Favorite	17.98	18.89	3.33	3.33	0	0	0	0	0	0	0	0	0	0	0	0	0	0
Maydameva (Melkoplodniy mestniy)	0	0	0	0	0	0	50.83	50.83	15	0	0	0	0	0	0	0	0	0
Noshpoti	0	0	0	0	0	0	20.83	20	11.67	0	0	0	0	0	0	0	0	0
Species	*Prunus domestica* (plum)																	
Local (**)	41.38	35.56	16.67	8.89	0	0	30.83	30.83	12.5	0	0	0	26.67	17.78	15	1.67	0	2.22
Sortovoy (***)	0	0	0	0	0	0	0	0	0	0	0	0	31.11	28.33	16.67	0.56	0	1.11

S1 Table. Main apple varieties of highly represented species in HG, and their uses by country. Varieties that were used by less than 10% of farmers in all countries were excluded from this table. The values in the column with frequencies refer to the total of respondents by country, not just the subset that claimed to have the species. in the same way the other columns are percentages referring to the total of the sample, not the fraction of people who indicated that they have the species or variety. In this way the frequencies are comparable at least within the single country. When the value of the columns home / sale / etc. coincides with Freq it means that all the owners of that variety use it for that purpose. Totals calculated across uses (home consumption, sale or gift) for a particular variety may exceed 100% since households use the same variety for more than one purpose. Forest (*): material derived from seeds/root suckers harvested in wild (forest); Local (**): traditional local varieties to which the farmers interviewed could not attribute a name; Sortovoy (***): unknown cultivated variety: improved varieties developed by the national formal breeding program and exotic varieties to which the farmers interviewed could not attribute a name.
