# Supplementary material for: Home gardens of Central Asia: Reservoirs of diversity of fruit and nut tree species
Source: PLoS One. 2022 Jul 28;17(7):e0271398. doi: 10.1371/journal.pone.0271398 (PMC9333230; doi:10.1371/journal.pone.0271398)
Supplement: S1 Questionnaire — (DOCX) [file pone.0271398.s006.docx]

Semi-structured Interviews with Local People who have Home Gardens: Fruit tree conservation and management practices

Objectives

1) To understand the domestication gradient from forest to farm for fruit and nut tree species, especially apple, apricot and walnut; and the degree to which wild germplasm is maintained in home gardens of villages near the wild populations

2) To understand the potential and challenges for maintenance of the wild populations

Sampling

Interviews will be conducted in Uzbekistan, Tajikistan and Kyrgyzstan.

Some villages where samples were collected for genetic and nutrient analysis will be revisited; selection of villages, among those sampled originally, is based on their proximity to wild populations.

In total 13 villages; 6 in Kyrgyzstan, 4 in Tajikistan and 3 in Uzbekistan.

| Country | Province | Village | Tree species of interest |
| --- | --- | --- | --- |
| Kyrgyz Republic |  |  |  |
|  |  |  |  |
|  |  |  |  |
|  |  |  |  |
|  |  |  |  |
|  |  |  |  |
| Tajikistan |  |  |  |
|  |  |  |  |
|  |  |  |  |
|  |  |  |  |
| Uzbekistan |  |  |  |
|  |  |  |  |
|  |  |  |  |

In each village, interviews will be conducted with local home garden managers according to the following criteria:

1. Begin by identifying the households that were previously sampled that can be revisited; i.e., is the person primarily involved with the home garden available for an interview?
2. Ask both men and women in the household whether they are involved in taking care of the home garden/orchard:
   - 1. Enquire about who is the primary manager (makes main decisions) of the home garden and/or orchard
     2. Enquire about who works in the home garden and/or orchard (Q16)
     3. If both women and men are involved, speak with the primary man in 50% of households and the primary woman in the other 50% of households.

N.B. We want to hear from the perspectives of all those who are knowledgeable about the management of orchards and home gardens. Although in some contexts men and/or women may be the more visible managers of orchards and home gardens, do not assume that women and/or men are not also involved in or knowledgeable about this matter.

When Interviewing

1. Make sure you ask the same questions to each participant since you will be comparing their responses. However, you may wish to change the sequencing of questions to improve the flow of the conversation. You may also probe more deeply on issues that emerge as important that may not be adequately captured in the interview guide.
2. Please do not ask participants to repeat themselves if they have already answered a question while responding to another question. It is your task to move the information around when filling out the database.
3. Give participants space to express themselves. If you do not agree with what they are saying, please do not challenge or contradict them. You may ask for more detailed explanations instead and try to understand their perspective and experiences. There are no right or wrong answers.
4. Do not interrupt people, but manage your time well so you do not run out of time to ask important questions. Bear in mind that participants will get tired if the interview is too long. Try to keep your interviews no longer than approximately one hour, unless the conversation is very engaging and you sense that the participant is keen to continue discussing with you. Be respectful of people’s time and other responsibilities.
5. It is a good idea to conduct interviews in participants’ home gardens. That way, participants can show you their trees and you can make observations of your own and ask them to comment on these. If the participant accepts, please take pictures of the garden, trees, participant and more, as relevant to the interview topic for use in your report.

When Note-taking

1) Careful note-taking is critical. To the fullest extent possible, document in people’s own words the reasons for their responses. Notes should be in the first person. It may be necessary to paraphrase especially long responses, but try to capture in full quotes the most important statements made by the informants.

2) Review your notes immediately after the interview or, if not possible, that very day. At that point, you will likely need to edit, expand, or provide explanatory comments about the interview. If too much time passes before you do this, valuable information will be lost due to notes that may only be partial, confusing or contradictory.

3) Add your own explanations, comments or observations but differentiate between these and participant responses by noting these down in the margins, in another font or colour.

Semi-structured Interviews with Local People who have Home Gardens

| **Participant Information**  Interview code ______  Village name: ________________________________________  Interviewer: ______________________________ Date: _________________________  Respondent’s name: ___________________________________________  Male ___ Female ___  Age: _____  Relationship to household head __________________________  Marital status _______________________  Level of education completed _________________  Number of children: _____  Primary occupation ______­­­­­­­­­­­___________________________  Secondary occupation _______________________________  Main ethnic group of the household ______________________  Length of time lived in the community _______ years |
| --- |

Household Roster

| ID | Name (first name) | Sex | Age | Relationship to HH head* | Marital status** | Education (primary/  secondary/higher) | Main occupation | Work in HG/orchard? (Yes/no) | Management/Decision-making over HG?  (1-3: 1=none, 2=some, 3=main) |
| --- | --- | --- | --- | --- | --- | --- | --- | --- | --- |
| 1 |  |  |  |  |  |  |  |  |  |
| 2 |  |  |  |  |  |  |  |  |  |
| 3 |  |  |  |  |  |  |  |  |  |
| 4 |  |  |  |  |  |  |  |  |  |
| 5 |  |  |  |  |  |  |  |  |  |
| 6 |  |  |  |  |  |  |  |  |  |
| 7 |  |  |  |  |  |  |  |  |  |
| 8 |  |  |  |  |  |  |  |  |  |
| 9 |  |  |  |  |  |  |  |  |  |
| 10 |  |  |  |  |  |  |  |  |  |

* (1) Self; (2) spouse; (3) son/daughter; (4) daughter or son in-law; (5) grandson/daughter; (6) father/mother; (7) brother/sister; (8) mother or father in-law; (9) sister or brother-in-law; (10) nephew/niece; (11) uncle/aunt; (12) other relative; (13) other (please specify)

** (1) Unmarried; (2) married; (3) widow/widower; (4) divorced/separated; (5) other

Semi-structured Interviews with Local People who have Home Gardens

**Interview Guide**

[Read:] Thank you for taking the time to talk with us today. We would like to ask you about how take care for your fruit and nut trees and how this may have changed over the years. This interview is part of a larger study being conducted with women and men here, in different villages in our country and in [Kyrgyzstan, Uzbekistan, Tajikistan]. We would like to learn about your experience and to have the knowledge you share with us today help us design better initiatives to support farmers/resource managers in this region in managing their fruit and nut trees in sustainable ways.

Your participation today is voluntary and confidential. I will not be using your name or the name of this community in any publication with the information that we collect today. I hope that you will feel free to express your opinions fully and share your own experiences with the topics that we will be discussing. You are of course free not to answer any question and to leave the discussion whenever you like. However, I very much hope you will remain for the entire discussion since your views and experiences are very important to me. I cannot promise that you and your community will benefit directly from this study, but the information that I am collecting is meant to help improve research and development activities on fruit trees in your country and in other Central Asian countries.

Do you agree to participate in the interview? _____ (Please check when informed consent is granted)

Are there any questions before we begin?

Part I: Description of the Home Garden and its Use

1. Can you tell me a little about your home garden? *This open ended question is a conversation starter. No need to spend much time here. The person may or may not have anything to say. If not, move on to question 2.*

__________________________________________________________________________________

__________________________________________________________________________________

__________________________________________________________________________________

__________________________________________________________________________________

1. How many trees are in your home garden?

__________________________________________________________________________________

1. Which types of trees do you grow here? (*Fill table below*)
   - 1. Which species?
     2. Which varieties?
     3. How many trees of each species and/or (iv) variety? *NB should add up to the total number of trees in the home garden. Please validate by asking, once the table is filled: “so you have X trees in total?”*
2. What do you use the fruits/nuts of the (i) species and/or (ii) varieties for (home consumption, sale, gifts, exchange, other (specify)? *More than one answer is possible. Please indicate all responses.*

| 3 i. Species | 3 iii. Number of trees | 4 i. Use |
| --- | --- | --- |
|  |  |  |
|  |  |  |
|  |  |  |
|  |  |  |
|  |  |  |
|  |  |  |
|  |  |  |
|  |  |  |
| **TOTAL** |  |  |

| 3 ii. Varieties | 3 iv. Number of trees | 4 ii. Use |
| --- | --- | --- |
|  |  |  |
|  |  |  |
|  |  |  |
|  |  |  |
|  |  |  |
|  |  |  |
|  |  |  |
|  |  |  |
| **TOTAL** |  |  |

1. If different varieties (of the same species) are used for home consumption and sale, why?

__________________________________________________________________________________

1. Do you also have a commercial orchard?

__________________________________________________________________________________

1. If so, what species and varieties do you grow there and how many of each?

*N.B. Make sure the distinction between a home garden and an orchard is clear to participants, who may grow fruit for both subsistence and sale on the same plot.*

Part II: Sourcing of Germplasm – Roles and Origins

1. Coming back to your home garden, when were the first trees in your home garden planted?

__________________________________________________________________________________

1. Did you plant them yourself (the first trees)? YES / NO If no, please explain.

__________________________________________________________________________________

1. Who (else) in your household, if anyone, has planted trees in your home garden? *This question should be adapted based on the previous answer: if respondent did not plant trees – who planted them? If respondent planted trees – who else plants? Apply IDs from household roster.*

__________________________________________________________________________________

1. What characteristics do you look for when you decide to plant certain trees from the forest or other sources? Taste qualities, or resistance to environmental stresses or market requirements, or any other specifies of the selected variety/form?

__________________________________________________________________________________

__________________________________________________________________________________

1. Who in your household decides to plant the trees? Is that a decision made individually or jointly, and by whom? *Apply IDs from household roster.*

__________________________________________________________________________________

1. Who acquired the planting material? *Match to household roster IDs – can be more than one ID.*

__________________________________________________________________________________

1. Where did the planting material come from? *E.g. forest, nurseries (which ones), family, Leskhoz, neighbours, etc. If different household members are sourcing this planting material from different origins, please make note of who is sourcing from where in parentheses, in the ‘origin of planting material’ cell.*
   - 1. *If more than one source*: Which of these sources do you get the greatest number of trees from (*please rank sources*)? Can you please rank those sources in order of importance (most important to least important)?

| **Origin of planting material** | i. **Rank** |
| --- | --- |
|  |  |
|  |  |
|  |  |
|  |  |

1. *Prompt more deeply about material obtained from the forest:* Do you ever obtain seed or seedlings (including rootstock) from nearby forests for this purpose? *This should validate responses to question 13.* YES / NO
   - 1. Why or why not?

__________________________________________________________________________________

- - 1. What exactly is collected (*seed, seedlings, rootstock, graft*)?

__________________________________________________________________________________

- - 1. What month(s) of year does this collection occur?

__________________________________________________________________________________

- - 1. Who gathers these? *Question 12 is similar, but refers to all sources of planting material; this one refers specifically to material obtained from the forest. This question can be used to validate the information gathered in Q12 if already answered.*

__________________________________________________________________________________

- - 1. Do you/does this person visit particular areas of the forest to gather these? YES / NO Please explain.

__________________________________________________________________________________

- - 1. Why or why not?

__________________________________________________________________________________

- - 1. Do/how do you/they select the specific seed or seedlings?

__________________________________________________________________________________

- - 1. What is your success rate in growing these (what proportion survives -- %)?

__________________________________________________________________________________

1. *Prompt about material obtained from other sources, based on response provided in question 14.* Do you get grafts or young trees that are not from the forest? *If no material is obtained from the forest, skip to question 16.* YES / NO
   - 1. What do you like about these trees?

__________________________________________________________________________________

- - 1. Do they present any inconveniences or drawbacks? YES / NO Please explain.

__________________________________________________________________________________

- - 1. Do you have any problems successfully establishing them? YES / NO Please explain.

__________________________________________________________________________________

- - 1. Do they survive as well as forest sources? *(if respondent also relies on forest sources)* YES / NO Please explain.

__________________________________________________________________________________

1. Do you ever give seedlings or material for grafting to anyone? YES / NO
   - 1. If so, to whom?

__________________________________________________________________________________

Part III: Collection from the Forest

1. Coming back to what you harvest from the forest: do you also collect fruits and nuts from the forest? YES / NO
   - 1. Which fruit or nuts?

__________________________________________________________________________________

- - 1. From which forest areas specifically?

__________________________________________________________________________________

- - 1. Who (not names) in your household harvests these? *Match ID to household roster.*

1. Do you also harvest trees for timber or construction? YES / NO
2. If so, how many per year?

__________________________________________________________________________________

1. Do you or someone in your household harvest hay from the forest? YES / NO
2. Have you ever planted or do you ever plant trees in the forest? YES / NO
   - 1. If so, which species?

__________________________________________________________________________________

- - 1. Why do you plant them in the forest?

__________________________________________________________________________________

- - 1. Where exactly do you plant them in the forest?

__________________________________________________________________________________

- - 1. What proportion of them survives?

__________________________________________________________________________________

- - 1. Where do you get the planting material for this?

__________________________________________________________________________________

1. Do you or anyone from your household ever protect seedlings (can be spontaneously grown or planted) in the forest from cattle or other livestock? YES / NO
   - 1. If so, who does this and where exactly? *Please match ID to household roster.*

__________________________________________________________________________________

Part IV: Silviculture and Future Vision

1. Coming back to your home garden now, how do you care for your trees?
2. Who, if anyone, in your household does the following?

| **Skills** | i. Yes/No | ii. Who has those skills? (*Match to household roster ID*) |
| --- | --- | --- |
| Selecting/collecting planting material |  |  |
| Planting |  |  |
| Growing seedlings |  |  |
| Pruning |  |  |
| Grafting |  |  |
| Managing tree health (against pathogens, etc.) |  |  |
| Watering |  |  |
| Harvesting fruit |  |  |
|  |  |  |
| Other (specify) |  |  |

1. Where did you learn to tend fruit or nut trees?

__________________________________________________________________________________

1. Are there any skills that you would like to have support in acquiring through trainings (e.g. grafting)? YES / NO If so, which?

__________________________________________________________________________________

- - 1. And if so, who in your household do you think should attend such trainings? *Match to household ID.* Why?

__________________________________________________________________________________

1. Do you think that you will continue to manage the fruit or nut trees in your home garden or orchard the same way in the future? YES / NO
   - 1. Why or why not?

__________________________________________________________________________________

1. Do you plan to plant more trees (establish an orchard or enlarge your orchard)? YES / NO

__________________________________________________________________________________

1. Why or why not?

__________________________________________________________________________________

1. If so where do you think you would get the trees?

__________________________________________________________________________________

1. Why from those sources?

__________________________________________________________________________________

1. You said earlier that your children *do/do not* help you in your home garden/orchard. Do you expect your children to stay in the village? YES / NO

__________________________________________________________________________________

Thank you for being so generous with your time today. Is there anything you would like to ask me before I go?

General comments from interviewer:
