# Supplementary material for: Home gardens of Central Asia: Reservoirs of diversity of fruit and nut tree species
Source: PLoS One. 2022 Jul 28;17(7):e0271398. doi: 10.1371/journal.pone.0271398 (PMC9333230; doi:10.1371/journal.pone.0271398)
Supplement: S2 Questionnaire — (DOCX) [file pone.0271398.s007.docx]

Полуструктурированные интервью с сотрудниками и руководителями лесных хозяйств: Практики сохранения и управления плодовыми и ореховыми деревьями

Цель

Понять потенциал и проблемы для поддержания популяций диких плодовых и ореховых деревьев.

Отбор образцов

Интервью будут проводиться в Узбекистане, Таджикистане и Кыргызстане.

Лесные хозяйства, где были отобраны образцы для генетического анализа и анализа питательных веществ, будут посещены повторно для целей исследования.

В общей сложности 20 лесных хозяйств; 7 в Кыргызстане, 7 в Таджикистане и 6 в Узбекистане, будут включены в исследование.

| Страна | Область | Лесное хозяйство | Интересующие виды деревьев |
| --- | --- | --- | --- |
| Кыргызская Республика |  |  |  |
|  |  |  |  |
|  |  |  |  |
|  |  |  |  |
|  |  |  |  |
|  |  |  |  |
|  |  |  |  |
| Таджикистан |  |  |  |
|  |  |  |  |
|  |  |  |  |
|  |  |  |  |
|  |  |  |  |
|  |  |  |  |
|  |  |  |  |
| Узбекистан |  |  |  |
|  |  |  |  |
|  |  |  |  |
|  |  |  |  |
|  |  |  |  |
|  |  |  |  |

Интервью будут проводиться с сотрудниками каждого лесхоза в соответствии с следующими критериями:

1. Включите представителей каждого уровня, т.е. директора, лесников, других работников
2. Если в любой из категорией заняты работники женщины, убедитесь в участии представителей каждого пола. Если в лесном хозяйстве работают только две или три женщины, интервью необходимо провести с каждой из них, чтобы получить мнение женщин.

При проведении интверью

1. Убедитесь, что вы задаете одни и те же вопросы каждому участнику, поскольку вы будете сравнивать их ответы. Тем не менее, вы можете изменить последовательность вопросов, чтобы улучшить ход беседы. Вы также можете более глубоко рассмотреть вопросы, которые оказываются важными, и которые могут не быть в полной мере охвачены руководством по проведению интервью.
2. Пожалуйста, не просите участников повторяться, если они уже ответил на вопрос, отвечая на другой вопрос. Это ваша задача управлять информацией при заполнении базы данных.
3. Дайте участникам пространство для самовыражения. Если вы не согласны с тем, что они говорят, пожалуйста, не оспаривайте их мнение и не противоречьте им. Вы можете попросить предоставить более подробные объяснений, и попытаться понять их точку зрения и опыт. Не существует правильных или неправильных ответов.
4. Не прерывайте людей, но управляйте своим временем, чтобы вам его хватило задать важные вопросы. Имейте в виду, что участники устанут, если интервью будет длиться слишком долго. Старайтесь чтобы ваши интервью не длились более приблизительно одного часа, если того разговор не является крайне интересным и вы чувствуете, что участник стремится продолжить обсуждение с вами. Уважайте время и другие обязанности людей.
5. Для получения информации о, как "официальном", так и "неофициальном" мнении о лесном хозяйстве, вы можете попросить о проведении интервью некоторых сотрудников лесного хозяйства в их офисах, при этом для интервью других сотрудников, вы можете пригласить их на чай или перекусить за пределами офиса, где атмосфера может быть более расслабленной. Убедитесь, что указываете в документах место проведения интервью. Рассмотрите просьбу к вашему собеседнику показать вам оборудование или помещения лесного хозяйства, если это уместно, и запишите свои наблюдения о них.

При ведении заметок

1) Тщательное ведение заметок имеет решающее значение. В максимально возможной степени, документируйте словами людей причины их ответов. Заметки должны вестись в первом лице. Возможно потребуется перефразировать особенно длинные ответы, но при этом попытаться отразить полными цитатами наиболее важные заявления информаторов.

2) Просмотрите свои заметки непосредственно после интервью, или, если это невозможно, в тот же день. В тот момент, вам, вероятно, потребуется отредактировать, расширить или предоставлять пояснительные комментарии по интервью. Если пройдет слишком много времени, прежде чем вы это сделаете, ценная информация будет потеряна, так как заметки могут быть лишь частичными, запутанными или противоречивыми.

3) Добавить свои собственные объяснения, комментарии или замечания, но различать из от ответов участников, записывая их на полях, или другим шрифтом или цветом.

Полуструктурированные интервью с сотрудниками лесных хозяйств

| **Информация об участнике**  Код интервью ______  Название лесного хозяйства: ______________________________________  Название деревни: ________________________________________  Интверьюер: ______________________________ Дата: _________________________  Ф.И.О респондента: ___________________________________________  Муж. ___ Жен. ___  Возраст: _____  Уровень законченного образования _________________  Основной род деятельности ______­­­­­­­­­­­___________________________  Дополнительный род деятельности _______________________________  Продолжительность периода проживания в сообществе _______ год / лет |
| --- |

Полуструктурированные интервью с сотрудниками лесных хозяйств

**Руководство по проведению интервью**

[Прочтите:] Спасибо, что нашли время, чтобы поговорить с нами сегодня. Мы хотели бы задать вам вопросы о местных практиках управления лесом и о том, как лес развивался с течением времени. Данное интервью является частью более крупного исследования, проводимого с местными жителями, а также лесоводами в различных деревнях в нашей стране и в [Кыргызстане, Узбекистане, Таджикистане]. Знания, которыми вы поделитесь с нами сегодня, помогут нам разработать более эффективные инициативы для поддержки фермеров / менеджеров ресурсов в этом регионе в управлении их плодовыми и ореховыми деревьями устойчивым образом.

Ваше участие сегодня является добровольным и конфиденциальным. Я не буду использовать ваше имя или название этого сообщества в какой-либо публикации с информацией, содержащей информацию, полученную сегодня. Я надеюсь, что вы будете свободно выражать свои мнения в полной мере и поделитесь своим опытом по темам, которые мы будем обсуждать. Вы, конечно, можете не отвечать на любой вопрос и покинуть обсуждение, когда вам захочется. Тем не менее, я очень надеюсь, что вы останетесь на период всего обсуждения, так как ваши мнения и опыт очень важны для меня. Я не могу обещать, что вы и ваше сообщество получите прямые выгоды от этого исследования, но информация, которую я собираю, предназначена для содействия улучшению научно-исследовательской работы и деятельности по развитию в сфере плодовых деревьев в вашей стране и в других странах Центральной Азии.

Вы все согласны участвовать в интервью? _____ (Пожалуйста, поставьте галочку, когда получите информированное согласие)

Есть ли у вас какие-либо вопросы, прежде чем мы начнем?

Часть I: Описание леса и его изменение со временем

1. Какова ваша роль в использовании и / или управлении лесом?

__________________________________________________________________________________

__________________________________________________________________________________

1. Можете ли вы рассказать мне, пожалуйста, немного о вашем лесе? Каким он был 15 лет назад?

__________________________________________________________________________________

__________________________________________________________________________________

__________________________________________________________________________________

__________________________________________________________________________________

1. И какой он сегодня?

__________________________________________________________________________________

__________________________________________________________________________________

__________________________________________________________________________________

__________________________________________________________________________________

1. Вы встречаете небольшие плодовые или ореховые деревья (сеянцы) в лесу?

__________________________________________________________________________________

__________________________________________________________________________________

- - 1. Если да, насколько они маленькие (ниже колена? от колена до талии, на уровне талии?..)

__________________________________________________________________________________

- - 1. Вы встречаете их в большом количестве или не нет?

__________________________________________________________________________________

__________________________________________________________________________________

- - 1. Зависит ли это от вида?

__________________________________________________________________________________

Часть II: Ощутимые угрозы лесу

1. Вы упомянули, что лес изменилось *X и Y образом* за последние 15 лет. Почему вы так думаете?
   - 1. По вашему мнению, какие самые большие угрозы для плодовых или ореховых деревьев в лесу? *Записывайте угрозы на листе бумаги в виде таблицы, по мере того, как их называют.*

| Угроза | Связана с конкретным видом? (ДА / НЕТ) | Категория | Что можно сделать, чтобы уменьшить угрозу? |
| --- | --- | --- | --- |
| Выпас скота |  |  |  |
| Производство топливной древесины |  |  |  |
| Сенокос |  |  |  |
| Другое (укажите) |  |  |  |
|  |  |  |  |
|  |  |  |  |
|  |  |  |  |
|  |  |  |  |

1. Зависит ли это от вида?
   - 1. Можете ли вы классифицировать эти угрозы в порядке важности, от самой важной (№1) к менее важной? *Добавьте крайнюю правую колонку, только когда вы дойдете до этого вопроса.*
2. Как вы думаете, урожайность некоторых видов увеличится или уменьшится в будущем? Если да, то у каких видов и почему? *Нарисуйте таблицу на листе бумаги с тремя крайними левыми колонками и заполняйте их систематически, по мере того, как даются ответы.*

| Вид | Урожайность увеличится / уменьшится | Причины | Качество фруктов / орехов повысится / снизится | Причины |
| --- | --- | --- | --- | --- |
|  |  |  |  |  |
|  |  |  |  |  |
|  |  |  |  |  |
|  |  |  |  |  |

- - 1. Вы думаете, что качество собранных фруктов и орехов конкретных видов повысится или снизится? Если да, то у каких видов и почему? *Добавьте две колонки справа, когда будет задан этот вопрос.*

1. Как вы думаете, скот наносит вред плодовому / ореховому лесу? *Если упоминалось выше, пожалуйста, задайте вопрос со слов "Вы упомянули, что крупный рогатый скот угрожает лесу, ..."*
2. Почему или почему нет?

__________________________________________________________________________________

__________________________________________________________________________________

__________________________________________________________________________________

__________________________________________________________________________________

1. Если да, почему допускает выпас скота в лесу?

__________________________________________________________________________________

__________________________________________________________________________________

__________________________________________________________________________________

1. Кто-нибудь выступает против такой практики?

__________________________________________________________________________________

__________________________________________________________________________________

__________________________________________________________________________________

1. Растет ли число скота, который использует лес, или остается примерно таким же, или снижается за последние 10 лет? Почему?

__________________________________________________________________________________

__________________________________________________________________________________

__________________________________________________________________________________

1. В какое время года скот пасется в лесу?

__________________________________________________________________________________

__________________________________________________________________________________

1. Растет ли продолжительность периода выпаса скота в лесу, или остается примерно такой же, или сокращается за последние 10 лет? Почему?

__________________________________________________________________________________

__________________________________________________________________________________

1. Где скот пасется остальное время?

__________________________________________________________________________________

__________________________________________________________________________________

Часть III: Передовые практики управления лесом

1. Как вы думаете, лесом хорошо управляют?
   1. Почему или почему нет?

__________________________________________________________________________________

__________________________________________________________________________________

__________________________________________________________________________________

__________________________________________________________________________________

1. По-вашему, какие передовые практики могли бы улучшить состояние леса? *Пожалуйста, ведите список на бумаге, добавляя столбцы по мере того, как задаются вопросы.*

| Передовая практика | Категория | Выполняется? | Почему? | Кем? | Выполнима? |
| --- | --- | --- | --- | --- | --- |
|  |  |  |  |  |  |
|  |  |  |  |  |  |
|  |  |  |  |  |  |
|  |  |  |  |  |  |

- - 1. Можете ли классифицировать эти практики в порядке важности, от наиболее важных до наименее важных, с точки зрения их потенциального воздействия на леса?
    2. Используются ли эти практики здесь, в деревне?
    3. Почему или почему нет?
    4. Как вы думаете, кто должен принять эти практики?
    5. Как вы думаете, они захотят или будут способны принять эти практики? Почему?

Дальнейшее изучение вопроса практик *(Пожалуйста, не просите людей повторять то, что уже было сказано):*

1. Осуществляются ли какие-либо мероприятия для защиты сеянцев в лесу от крупного рогатого скота или других домашних животных?

__________________________________________________________________________________

__________________________________________________________________________________

__________________________________________________________________________________

1. Как вы думаете, жители могут принять такие практики?

__________________________________________________________________________________

1. Почему или почему нет?

__________________________________________________________________________________

__________________________________________________________________________________

__________________________________________________________________________________

__________________________________________________________________________________

1. Кто-нибудь (сотрудники лесного хозяйства или жители деревни) сажает деревья в лесу?

__________________________________________________________________________________

- - 1. Если да, то кто (без указания имен)?

__________________________________________________________________________________

__________________________________________________________________________________

- - 1. Какие виды растений они сажают?

__________________________________________________________________________________

__________________________________________________________________________________

_________________________________________________________________________________

- - 1. В каком количестве?

__________________________________________________________________________________

__________________________________________________________________________________

__________________________________________________________________________________

- - 1. Осуществляются ли какие-либо мероприятия для обеспечения выживания этих сеянцев - и выживают ли они?

__________________________________________________________________________________

__________________________________________________________________________________

__________________________________________________________________________________

- - 1. Что делается?

__________________________________________________________________________________

__________________________________________________________________________________

__________________________________________________________________________________

- - 1. Кем (без указания имен)?

__________________________________________________________________________________

__________________________________________________________________________________

__________________________________________________________________________________

1. Кто-нибудь прививает деревья в лесу?

__________________________________________________________________________________

- - 1. Кто делает это (без указания имен)?

__________________________________________________________________________________

__________________________________________________________________________________

- - 1. Где именно?

__________________________________________________________________________________

- - 1. Почему?

__________________________________________________________________________________

__________________________________________________________________________________

1. Есть ли вашем лесном хозяйстве люди, которые особенно внимательно заботятся о лесе?

__________________________________________________________________________________

__________________________________________________________________________________

- - 1. Если да, то кто они (пожилые люди,мужчины, женщины, молодые люди)?

__________________________________________________________________________________

__________________________________________________________________________________

- - 1. Что они делают особенно хорошего для леса?

__________________________________________________________________________________

__________________________________________________________________________________

- - 1. Является ли участие местных жителей достаточным в мероприятиях, связанных с защитой / сохранением ореховых и фруктовых деревьев в лесу? Если оно недостаточно, как оно может быть улучшено или усилено?

__________________________________________________________________________________

__________________________________________________________________________________

__________________________________________________________________________________

1. Какой уровень участия местного административного совета (айыл окмоту или бывший сельсовет) в распределении билетов (разрешений) на выпас скота, производство дров и сенокос?

__________________________________________________________________________________

__________________________________________________________________________________

1. При принятии решений, лесное хозяйство должно консультироваться с административным советом деревни (айыл окмоту или бывший сельсовет) и учитывать его мнение?

__________________________________________________________________________________

__________________________________________________________________________________

__________________________________________________________________________________

__________________________________________________________________________________

__________________________________________________________________________________

Часть IV: Видение будущего

1. Как вы думаете, лес будет продолжать производить фрукты и орехи, когда будут жить поколения ваших детей и внуков?
   - 1. Почему или почему нет?

__________________________________________________________________________________

__________________________________________________________________________________

__________________________________________________________________________________

__________________________________________________________________________________

Спасибо за то, что уделили нам свое время сегодня. Хотите ли вы задать мне какие-либо вопросы, прежде чем я уйду?
